# Novel Antidepressant-Like Properties of the Iron Chelator Deferiprone in a Mouse Model of Depression

**DOI:** 10.1007/s13311-022-01257-0

**Published:** 2022-07-21

**Authors:** Volkan Uzungil, Harvey Tran, Connor Aitken, Carey Wilson, Carlos M. Opazo, Shanshan Li, Jennyfer M. Payet, Celeste H. Mawal, Ashley I. Bush, Matthew W. Hale, Anthony J. Hannan, Thibault Renoir

**Affiliations:** 1 Melbourne Brain Centre, Florey Institute of Neuroscience and Mental Health, University of Melbourne, Parkville, Australia; 2 School of Psychology and Public Health, La Trobe University, Melbourne, VIC 3086 Australia; 3 Faculty of Medicine, Dentistry and Health Sciences, University of Melbourne, Parkville, Australia; 4 Melbourne Dementia Research Centre, Florey Institute of Neuroscience and Mental Health, University of Melbourne, Parkville, Australia

**Keywords:** Antidepressant, Iron, Stress, Functional network

## Abstract

Depressed individuals who carry the short allele for the serotonin-transporter-linked promotor region of the gene are more vulnerable to stress and have reduced response to first-line antidepressants such as selective serotonin reuptake inhibitors. Since depression severity has been reported to correlate with brain iron levels, the present study aimed to characterise the potential antidepressant properties of the iron chelator deferiprone. Using the serotonin transporter knock-out (5-HTT KO) mouse model, we assessed the behavioural effects of acute deferiprone on the Porsolt swim test (PST) and novelty-suppressed feeding test (NSFT). Brain and blood iron levels were also measured following acute deferiprone. To determine the relevant brain regions activated by deferiprone, we then measured c-Fos expression and applied network-based analyses. We found that deferiprone reduced immobility time in the PST in 5-HTT KO mice and reduced latency to feed in the NSFT in both genotypes, suggesting potential antidepressant-like effects. There was no effect on brain or blood iron levels following deferiprone treatment, potentially indicating an acute iron-independent mechanism. Deferiprone reversed the increase in c-Fos expression induced by swim stress in 5-HTT KO mice in the lateral amygdala. Functional network analyses suggest that hub regions of activity in mice treated with deferiprone include the caudate putamen and prefrontal cortex. The PST-induced increase in network modularity in wild-type mice was not observed in 5-HTT KO mice. Altogether, our data show that the antidepressant-like effects of deferiprone could be acting via an iron-independent mechanism and that these therapeutic effects are underpinned by changes in neuronal activity in the lateral amygdala.

**Supplementary Information:**

The online version contains supplementary material available at 10.1007/s13311-022-01257-0.

## Introduction

Current antidepressants have limited efficacy, with a third of patients with major depressive disorder (MDD) presenting refractory depression to monoaminergic antidepressants [1]. In addition, typical antidepressant treatments can take up to 8 weeks before any substantial benefits are observed [2]. Newer classes of fast-acting antidepressants such as ketamine have been shown to have rapid actions within an hour and have approximately 70% efficacy in individuals with treatment-resistant depression [3, 4]. However, due to the psychomimetic, cognitive and dissociative effects of ketamine, there is an urgent need to discover better fast-acting antidepressants [5, 6].

There is both clinical and preclinical evidence which suggests that iron plays a role in cognitive and emotional states [7]. For instance, a quantitative susceptibility mapping study has found that depression severity was related to greater levels of iron in multiple brain regions of patients with MDD [8]. In a population of thalassemia patients which have elevated levels of the iron storage protein ferritin, there was an increased prevalence of depressive symptoms [9]. Iron also plays a role in monoamine neurotransmitter function as it is a co-factor for the production of neurotransmitters, including serotonin [10]. Iron has also been implicated in the production of reactive oxygen species [11] which play a role in MDD pathophysiology [12, 13]. Deferiprone is an iron chelator used therapeutically to treat transfusion iron overload and can rapidly cross the blood–brain barrier [14, 15].

In the present study, the serotonin transporter knockout (5-HTT KO) mouse of model of depression was used to test for the potential therapeutic actions of deferiprone. 5-HTT KO mice have been shown to display depression-related behaviours as well as altered responses to stressors [16–18]. There is evidence for the role of polymorphisms of the 5-HTT gene in depression [19, 20]. For instance, individuals who are carriers of the short (s) allele of the polymorphic region (5-HTTLPR) of the gene show alterations in stress tolerability, a key feature in MDD pathophysiology [21, 22]. A further utility of the 5-HTT KO mice is that it can be considered as a model of antidepressant treatment resistance, as both individuals who carry the s allele of the 5-HTTLPR and the 5-HTT KO mouse have reduced response to selective serotonin reuptake inhibitors [18, 23, 24]. The utility of the 5-HTT KO mouse model of depression to assess antidepressant properties of an iron chelator is indicated by the fact that selective serotonin reuptake inhibitor (SSRI) drugs were shown to reduce levels of peripheral haemoglobin, which is associated with levels of circulating iron [25]. In addition, 5-HTT KO mice have increased expression of oxidative stress markers [26].

Brain-wide regions are involved in depression and stress response, including the neocortex, the limbic system and the midbrain [27–29]. Concurrently, there is evidence that antidepressants act over multiple brain regions to mediate their therapeutic actions [30–35]. Selective regions which have been shown to be altered by 5-HTT function include the amygdala and dorsal raphe which have altered morphology and connectivity in the 5-HTT KO mice, as well as the lateral septum where 5-HTT availability plays a role in the acute stress response [36–39]. Furthermore, the therapeutic actions of antidepressants involve functional network-wide activities, shown to be altered in MDD [40, 41]. Network modularity has also been shown to be altered in psychiatric disorders and in response to acute stress [42, 43].

Using relevant behavioural tests, the current study explored the potential fast-acting antidepressant-like properties of acute administration with deferiprone in the 5-HTT KO mouse model of depression. Furthermore, the study examined the brain regions potentially mediating the antidepressant-like effects of deferiprone as well as regions implicated in the aberrant stress response in the 5-HTT KO mice. Finally, we applied graph theory-based and hierarchical clustering analyses to determine the functional architecture following deferiprone treatment and swim-stress exposure.

## Methods

### Animals and Housing

All animal care and procedures were approved by the Florey Institute of Neuroscience and Mental Health (FINMH) Animal Ethics Committee and conducted in concordance with guidelines published by the National Health and Medical Research Council (NHMRC). Male and female wild-type (WT) control and 5-HTT KO mice were bred on a C57Bl/6 J genetic background from a colony established at FINMH [18, 44]. Post-weaning, mice were housed [3–5 mice per cage] by littermates in individually ventilated cages until 8 weeks of age. At 8 weeks, they were transferred to open top cages (34 × 16 × 16 cm) with standard wood shaving bedding and maintaining original cage-mates. The animals had access to food and water ad libitum while housed in 12/12-hour light/dark cycle rooms (light at 07:00–19:00). All handling or behavioural experiments were done in the light phase. All experiments were conducted between 10 and 20 weeks of age. All experiments were conducted with the experimenter blind to genotype and treatment.

### Pharmacological Treatments

The iron chelator 3-hydroxy-1,2-dimethyl-4(1H)-pyridone (deferiprone; Sigma-Aldrich, Castle Hill, Australia) and 0.9% sterile saline/vehicle (Baxter, Old Toongabbie, Australia) were injected via intraperitoneal (i.p.) administration. Deferiprone was dissolved in 0.9% saline. A clinically relevant dose of 50 mg/kg deferiprone was selected [15]. To ensure there were no long-term effects of deferiprone treatment confounding the subsequent behavioural paradigm, mice were alternated between receiving either deferiprone or vehicle in each behavioural test.

### Porsolt Swim Test

The Porsolt swim test (PST) was conducted 1 hour after vehicle or deferiprone administration. In a separate cohort, the PST was also conducted 24 hours post-vehicle or deferiprone administration. The PST has been widely used to screen novel compounds for potential antidepressant activity, as well as studying acute stress-coping behaviour of rodents [18]. Each mouse was placed in a 2-L beaker filled with 1.5 L of water so that the mouse could not escape and the tail did not touch the bottom of the beaker. The mouse spent 6 minutes in the water with the last 4 minutes being used to determine immobility time as identified by floating behaviour. The temperature of the water was adjusted to 25 $\pm$ 1 ℃. The recording was analysed using the ForcedSwimScan (Clever Sys, Restin, USA) to determine total immobility time.

### Novelty-Suppressed Feeding Test

The novelty-suppressed feeding test (NSFT) is a conflict approach-avoidance paradigm in which antidepressants have been shown to reduce the latency to feed [45]. The testing arena (80 × 680 × 680 cm) contained bedding material in a low-lit room to reduce external stressors. In the centre of the arena, a white filter paper containing a food pellet was positioned. All mice were food deprived for 24 hours before the start of the test. On the testing day, the mouse was placed in the corner of the chamber 1 hour after receiving either vehicle or deferiprone treatment. They were then removed from the chamber either after consuming the food pellet or after 600 seconds had elapsed. Mice were then placed in their home cage for 300 seconds and the amount of pellet consumed was recorded.

### Locomotor Activity

To assess locomotor activity following deferiprone treatment, mice were first habituated to the photo-beam activity chamber (26 × 26 × 38 cm) (Coulburn Instruments, Pennsylvania, USA) for 30 minutes. Following habituation, the mice were injected with either vehicle or deferiprone and their locomotor activity for the next 60 minutes was then measured in the chamber.

### Inductively Coupled Plasma Mass Spectrometry (ICP-MS)

To assess for the acute effect of deferiprone treatment on metal levels, whole brain tissue or peripheral blood was collected. 1 hour following injection with deferiprone or vehicle, mice were anaesthetised with pentobarbital (80 mg/kg; Virbac, Milperra, Australia) in 0.9% saline i.p. Blood was removed via transcardial puncture following by perfusion with phosphate buffered saline (PBS, 0.05 M, pH 7.4) and brain regions of interest dissected. Blood collected in tubes was allowed to clot for 30–45 minutes at room temperature and then centrifuged for 15 minutes at 1100 × *g*. The serum from the sample was collected and stored at −20 °C until subsequent analysis. The brain tissue was frozen at −80 °C for storage.

During ICP-MS, the samples were first freeze dried before being digested. To the lyophilised tissue samples, 50 µL of nitric acid (HNO_3_) (65% Suprapur, Merck) was added and allowed them to digest overnight at room temperature. The samples were further digested by heating at 90 °C for 20 minutes using a heating block. Samples were then removed from the heating block and an equivalent volume of 50 µL hydrogen peroxide (H_2_O_2_) (30% Aristar, BDH) was added to each sample. Samples were allowed to stop effervescing, for 30 minutes, before heating again for a further 15 minutes at 70 °C. The average reduced volume was determined, and the samples were further diluted with 1% HNO_3_ diluent.

Measurements were made using an Agilent 7700 series ICP-MS instrument under routine multi-element operating conditions using a helium reaction gas cell. The instrument was calibrated using 0, 5, 10, 50, 100 and 500 ppb of certified multi-element ICP-MS standard calibration solutions (ICP-MS-CAL2-1, ICP-MS-CAL-3 and ICP-MS-CAL-4; Accustandard) for a range of elements. A certified internal standard solution containing 200 ppb of yttrium (Y89) was used as an internal control. Results are expressed as micrograms of metal per gram of wet weight tissue (μg/g) or micromoles of metal per litre of blood serum (μmol/L).

### Immunohistochemistry

To determine neuronal activity following exposure to deferiprone and also swim stress, c-Fos expression was measured following deferiprone treatment and/or swim stress/PST exposure. 90 minutes following the cessation of the swim stress, or 160 minutes after injection with deferiprone or vehicle, the mice were anaesthetised with pentobarbital (80 mg/kg; Virbac) in 0.9% saline i.p. After the mice were anaesthetised, they were transcardially perfused with phosphate buffered saline (PBS, 0.05 M, pH 7.4) followed by 4% paraformaldehyde (PFA) solution (0.1 M NaP buffer, 1.5% sucrose, 4% paraformaldehyde; Sigma-Aldrich, Castle Hill, Australia; pH 7.4) overnight. Following this, the brain was placed in 30% sucrose in 0.1 M phosphate buffer (PB) until the brain was sunk. Brains were fast frozen using dry ice submerged in isopentane and then stored in a −80 °C freezer. Coronal sections. (30 µm) were then cut for the whole brain using a cryostat into 24-well tissue culture plates and stored in a −20 °C freezer in cryoprotectant (30% ethylene glycol, 20% glycerol in 0.05 M NaP buffer; pH 7.4) solution. Peroxidase immunohistochemistry for c-Fos expression analysis was then conducted as previously described [46].

When conducting the immunohistochemistry staining, on the first day, sections were washed in 0.05 M PBS and then quenched for endogenous peroxidase in 1% H_2_O_2_ in 50% MeOH/50% 0.05 M PBS for 30 minutes. Sections were then washed and pre-incubated in 0.3% PBST (0.3% Triton X100) for 30 minutes. Sections were then placed in 5% normal goat serum (Thermo Fisher; in 0.3% PBST) for 60 minutes and finally incubated overnight in primary antibody (rabbit anti-c-Fos 1:3000; Millipore, Temecula, USA; CAT# ABE457, LOT# 3,168,266) in 0.3% PBST. On the second day, the sections were washed and then incubated in the secondary antibody (biotinylated-goat anti-rabbit 1:500; Vector Laboratories; CAT# BA-1000) in 0.05 M PBS for 90 minutes. The sections were then washed again and transferred to avidin–biotin-peroxidase complex (1:200; Vector Elite; CAT# PK-6101, LOT# ZF0425) for 90 minutes. Tissue sections were washed again and then stained in peroxidase chromogen solution according to manufacturer’s instructions (Vector SG; CAT# SK-4700) for 10 minutes. Sections were then mounted on slides and, once dry, were hydrated for 2 minutes in tap water and then progressively dehydrated in increasing concentrations of ethanol baths (50%, 90%, 2 × 100%) and xylene solution for 3 minutes followed by cover-slipping using DPX (Thermo Fisher, Leicestershire, UK).

For each region captured, the image was taken at the same bregma point for each mouse as identified in Supplementary Table 2. For each region, one brain section per animal was analysed. Photomicrographs were taken using a Zeiss Imager M2 microscope using a × 20 objective lens. The entire area of each region was captured through intervals using tiles and stitching functions of the Stereo Investigator 2019.1.2 software. The region of interest was determined via the mouse brain atlas (Paxinos & Franklin, 2010). Quantification of Fos-positive immunoreactive cells was recorded from a single hemisphere unless a region crossed into both hemispheres. Fos-positive stained cells were counted using a script on ImageJ and validated to manual counts. Cell count density was determined by Fos-positive immunoreactive cells divided by the area of region of interest.

### Functional Network Analyses

Graph theory-based analyses were applied to the c-Fos expression data to characterise the functional connectome [47, 48]. Correlation matrices were created for visualising inter-regional correlation. The matrices were created using Pearson’s correlation coefficient. Circular graphs representing significant nodes and edges in the functional connectome were created using significance set at Pearson’s *r* ≥ 0.83 for positive correlation and *r* ≤  −0.83 for negative correlation [48]. Nodes represent individual brain regions of interest while edges represent inter-regional correlation between two brain regions. Nodes were considered a hub, which is a key region for information integration in a network, if they were in the 80th percentile for degree and betweenness centrality as well as showing significance at the low (*r* ≥ 0.79), primary (*r* ≥ 0.83) and high (*r* ≥ 0.87) positive correlation confidence networks [48]. Community detection analysis was conducted using a spectral community detection algorithm which was applied to the weighted correlation network [49]. The community detection analysis maximises within-module correlation while minimising between-module correlation to group regions based on shared co-activity patterns, which are depicted as different colours in the network map. In each group, all amygdala regions taken from −1.82 bregma point were highlighted in bold to characterise their organisation in the community detection network. Rubinov and Spears Brain Connectivity Toolbox was employed for graph theory analysis using Matlab R2019b [50]. Visualisations were created using R v3.6.1 and packages ggplot2 and ggnet2.

Hierarchical clustering has been previously adopted to cluster regions of similar co-activation profile using c-Fos expression correlation datasets [42]. The tiles in each map represent all functional connections between the 60 brain regions analysed. Distance matrices have been hierarchically clustered to characterise modular structuring of functional brain networks [42, 51]. The Euclidean distance matrix was created for the inter-regional c-Fos expression correlation dataset. The distance matrix was hierarchically clustered using the complete method to identify modules in each group. The hierarchical cluster dendrogram was then tree cut at 70% of the height to determine the number of modules, as this height was representative of clustering at various cuts of the dendrogram and did not result in excessive modularity. The number of modules created at this tree cut was representative of the number of modules at various other values. Visualisations were created using R v3.6.1 and packages ComplexHeatmap.

### Data and Statistical Analysis

Data were analysed by a two-way analysis of variance (ANOVA) to look at the genotype × treatment, genotype × drug or genotype × stress interaction between factors. Data were analysed as a three-way ANOVA to look at the interaction between factors for the c-Fos expression dataset. Repeated-measures ANOVA was adopted if there were multiple time points or measurements repeated within subjects and measured from time of treatment. For the NSFT, a logrank Mantel–Cox test was adopted to evaluate differences between experimental groups. Sexes were pooled as no sex differences were observed in any of the tests. Significance was set at *p* < 0.05 and a Bonferroni post hoc test was adopted to calculate for pairwise comparisons. Statistical analysis was conducted using GraphPad prism 9.0 (GraphPad Software Inc., LA Jolla, CA, USA) and SPSS 22.0 (IBM, Armonk, NY, USA) software. Data are available on request from the authors. Scripts used for analysis can be found at https://github.com/vuzungil01/dfp_network.

## Results

### Deferiprone Has Acute Antidepressant-Like Effects

**Fig. 1:**
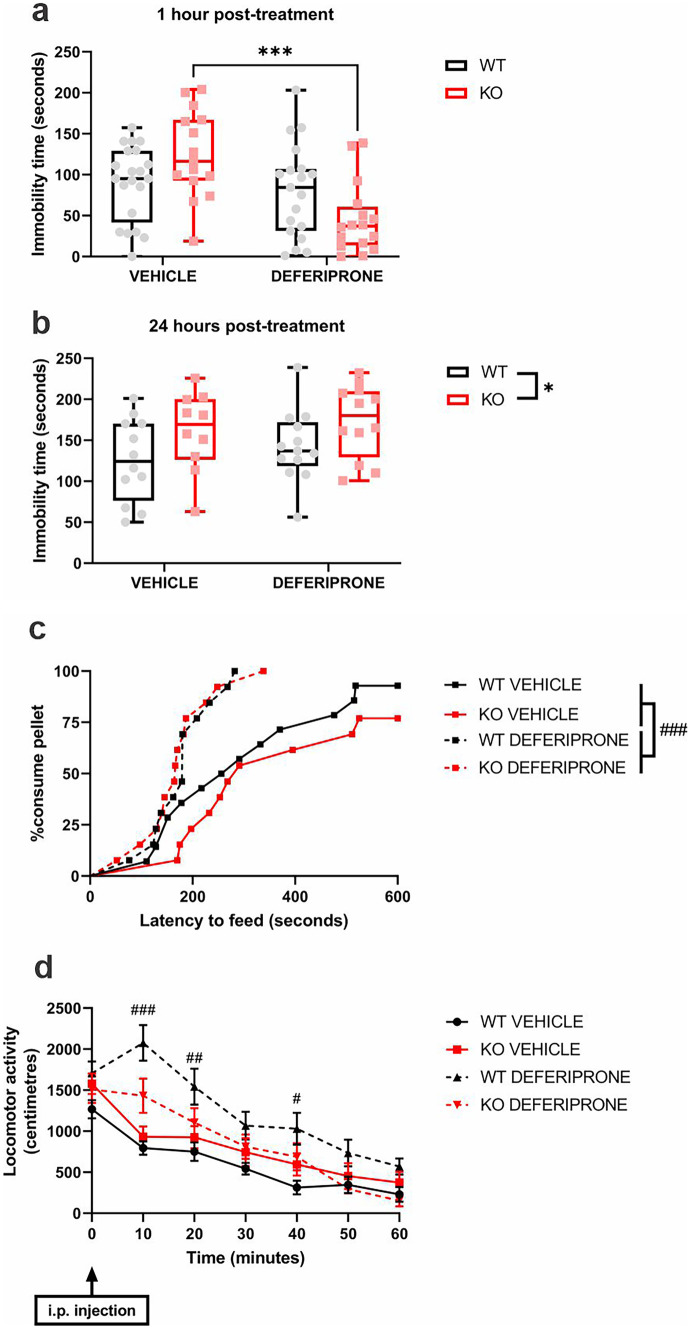
Effect of acute deferiprone on depression-related behaviours and locomotor activity. Effect of deferiprone on the Porsolt swim test at 1 hour (**a**) and 24 hours (**b**) post-injection (2-way ANOVA; Bonferroni post hoc). **c** Effect of deferiprone on the novelty-suppressed feeding test (Cox-regression test). **d** Locomotor activity in the 60 minutes following deferiprone injection (3-way repeated measure ANOVA; Bonferroni post hoc). **a, b** Data are expressed as median with interquartile range; whiskers represent min to max values. **d** Data are expressed as mean ± SEM. **a** *n* = 15–21; **b**, **d** *n* = 10–13; **c** *n* = 13–14. ***p < 0.001 KO vehicle vs. KO deferiprone, $*p* < 0.05 WT vs. KO, #*p* < 0.05; ##*p* < 0.01; ###*p* < 0.001 vehicle vs. deferiprone. WT = wild-type; KO = knock-out

The Porsolt swim test (PST) and novelty-suppressed feeding test (NSFT) are widely used to assess the potential antidepressant properties of drugs. In the PST, 60 minutes post-injection, deferiprone reduced immobility time in 5-HTT KO mice only (genotype × treatment interaction−*F*_(1,67)_ = 8.44, *p* < 0.05; post hoc−*p* < 0.001) (Fig. 1a). There was no effect of deferiprone at 24 hours post-injection (treatment—*F*_(1,43)_ = 0.022, *p* > 0.05), but 5-HTT KO mice had increased immobility compared to WT littermate controls (genotype−*F*_(1,43)_ = 5.82, *p* < 0.05) (Fig. 1b). Antidepressant-like activities in the NSFT were found, as deferiprone-treated mice had reduced latency to feed in both genotypes (treatment−hazard ratio = 3.98, *p* < 0.001) (Fig. 1c). Since 5-HTT KO mice are also considered a model of anxiety [52], the effect of acute deferiprone treatment was also assessed in the light–dark box, in which the drug reduced the time spent in the light compartment (Fig. S1). Ruling out any potential locomotor confounding effect in the PST and NSFT findings, there was no effect of deferiprone in KO mice 60 minutes post-injection, as deferiprone only increased locomotor activity in both genotypes at 10, 20 and 40 minutes post-injection compared to their vehicle counterparts (time × treatment interaction−*F*_(4.86, 199.13)_ = 6.46, *p* < 0.001; post hoc = 10 minutes (*p* < 0.001), 20 minutes (*p* < 0.01) and 40 minutes (*p* < 0.05)) (Fig. 1d).

### Acute Deferiprone Treatment Had No Effect on Iron Levels in the Prefrontal Cortex, Striatum and Brainstem, or Peripheral Blood

**Fig. 2:**
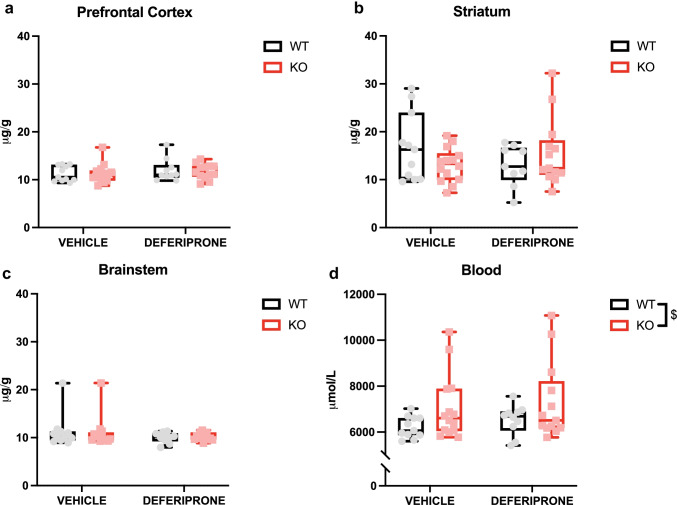
Levels of iron in brain regions and blood following acute deferiprone treatment. Effect of acute deferiprone treatment on the **a** prefrontal cortex, **b** striatum, **c** brainstem and **d** blood. Two-way ANOVA. Data are expressed as median with interquartile range; whiskers represent min to max values. *n* = 9–14. $*p* < 0.05 WT vs. KO overall effect. WT = wild-type; KO = knock-out

Levels of iron in various brain regions and blood were measured following acute deferiprone treatment (Fig. 2). There was no effect of deferiprone in the prefrontal cortex (*F*_(1,44)_ = 0.66, *p* > 0.05) (Fig. 2a), striatum (*F*_(1,43)_ = 1.38, *p* > 0.05) (Fig. 2b), brainstem (*F*_(1,43)_ = 2.32, *p* > 0.05) (Fig. 2c) and blood (*F*_(1,44)_ = 0.61, *p* > 0.05) (Fig. 2d). However, in blood, 5-HTT KO mice had elevated levels of iron compared to WT controls (*F*_(1,44)_ = 5.06, *p* < 0.05).

### Effects of Deferiprone Implicates the Lateral Amygdala, Dorsal Raphe and Lateral Septum in a Genotype-Specific Manner

**Fig. 3:**
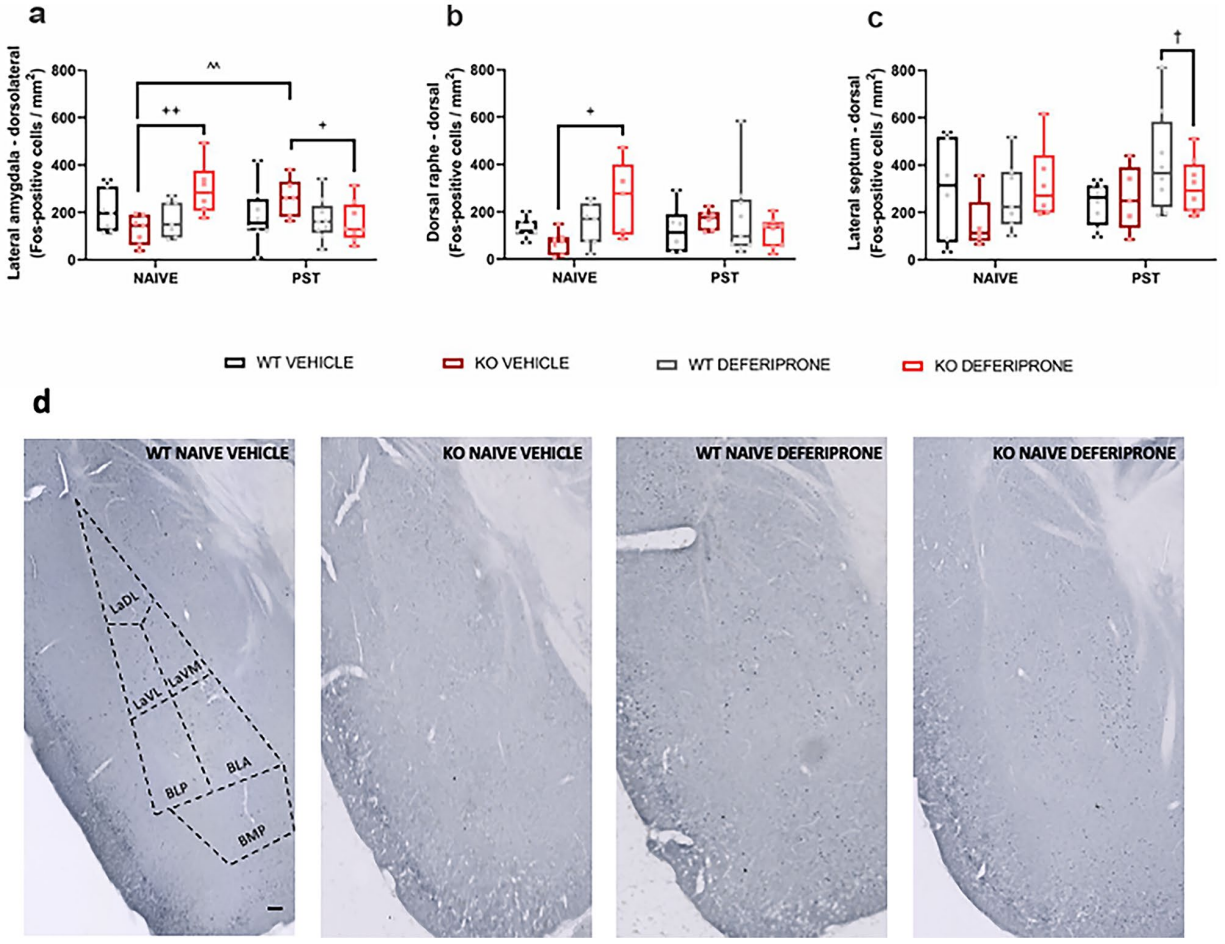
Acute deferiprone and swim stress exposure on c-Fos expression in various brain regions. Effect of deferiprone and swim stress exposure on c-Fos expression in subnuclei of the **a** lateral amygdala, **b** dorsal raphe and **c** lateral septum. **d** Representative photomicrographs of deferiprone treatment on behaviourally naive WT and 5-HTT KO mice in the basolateral, basomedial and lateral amygdala. Three-way ANOVA; Bonferroni post hoc. Data are expressed as median with interquartile range; whiskers represent min to max values. Black bar represents 100 μm. **a** *n* = 6–9, **b** *n* = 5–9, **c** *n* = 5–8. ^^*p* < 0.01 KO naive vs. KO PST, + *p* < 0.05; +  + *p* < 0.01 KO vehicle vs. KO deferiprone, † WT deferiprone vs. KO deferiprone, ##*p* < 0.01 vehicle vs. deferiprone. WT = wild-type; KO = knock-out; BNST = bed nucleus of the stria terminalis; PST = Porsolt swim test; LaDL = lateral amygdala−dorsolateral; LaVL = lateral amygdala−ventrolateral; LaVM = lateral amygdala−ventromedial; BLP = basolateral amygdala−posterior; BLA = basolateral amygdala−anterior; BMP = basomedial amygdala−posterior

The present study next aimed to determine which regions were implicated in the reduction of immobility behaviour of the 5-HTT KO mice treated with deferiprone following swim stress/PST exposure (Fig. 3). This was done by assessing c-Fos expression as a marker of neuronal activity following deferiprone treatment and swim stress exposure. c-Fos expression was also used to determine brain-wide effects of deferiprone in both mice naive of behavioural testing as well as following swim stress exposure (Table 1).

**Table 1:** Density of Fos-positive cells in brain regions following deferiprone treatment in both WT and 5-HTT KO mice, behaviourally naive and following swim stress

Brain regions (distance from bregma, mm)	WT Naive	KO Naive	WT Naive	KO Naive	WT PST	KO PST	WT PST	KO PST
	Vehicle	Vehicle	Deferiprone	Deferiprone	Vehicle	Vehicle	Deferiprone	Deferiprone
**Prefrontal cortex**								
**Prelimbic cortex (+ 1.70)**	357 ± 81	288 ± 38	358 ± 72	463 ± 77	327 ± 34	371 ± 67	335 ± 29	317 ± 41
**Infralimbic cortex (+ 1.70)**	238 ± 53	208 ± 42	326 ± 52^##^	402 ± 38^##^	316 ± 19**	362 ± 34**	336 ± 40	335 ± 33
**Frontal association cortex (+ 2.58)**	44 ± 10	70 ± 27	33 ± 12	80 ± 15	63 ± 18	71 ± 31	69 ± 17	87 ± 19
**Medial orbital cortex (+ 2.58)**	258 ± 73	212 ± 27	187 ± 47	290 ± 50	348 ± 96	326 ± 77	280 ± 46	248 ± 50
**Ventral orbital cortex (+ 2.58)**	340 ± 85	325 ± 64	210 ± 57	367 ± 79	308 ± 69	377 ± 79	280 ± 62	284 ± 38
**Lateral orbital cortex (+ 2.58)**	261 ± 65	267 ± 47	166 ± 39	286 ± 66	262 ± 42	230 ± 36	226 ± 44	258 ± 37
**Dorsolateral orbital cortex (+ 2.58)**	110 ± 35	152 ± 33	156 ± 43	288 ± 74	164 ± 39	126 ± 28	142 ± 23	228 ± 66
**Cerebral cortex**								
**Cingulate cortex (+ 1.10)**	395 ± 51	302 ± 44	313 ± 43	344 ± 41	330 ± 39	285 ± 32	281 ± 40	297 ± 53
**Claustrum (+ 1.10)**	212 ± 37	128 ± 61	181 ± 44	212 ± 90	161 ± 42	153 ± 38	153 ± 37	212 ± 35
**Agranular insular cortex (+ 1.10)**	171 ± 38^#$^	75 ± 15	96 ± 16	133 ± 32^$^	171 ± 35^#$^	152 ± 30	107 ± 25	188 ± 26^$^
**Dysgranular insular cortex (+ 1.10)**	120 ± 22	77 ± 17	146 ± 37	155 ± 34^#^	123 ± 25	118 ± 27	105 ± 24^$^	246 ± 43^#$^
**Granular insular cortex (+ 1.10)**	140 ± 54	54 ± 11	187 ± 48^##a^	189 ± 50^##a^	110 ± 16	84 ± 17	158 ± 53^##a^	208 ± 32^##a^
**Lateral septum**								
**Lateral septum, dorsal (+ 0.50)**	300 ± 86	151 ± 52	272 ± 55	322 ± 66	238 ± 32	260 ± 61	413 ± 76^†^	309 ± 41
**Lateral septum, ventral (+ 0.50)**	339 ± 66	436 ± 106	478 ± 46	413 ± 56	503 ± 68	585 ± 67	395 ± 61	445 ± 77
**Lateral septum, intermediate (+ 0.50)**	278 ± 50	214 ± 45	309 ± 30	279 ± 54	341 ± 49^*a^	433 ± 73^*a^	355 ± 65^*a^	298 ± 37^*a^
**Hypothalamus**								
**Paraventricular hypothalamus (-1.06)**	284 ± 224	336 ± 155	446 ± 179	447 ± 223	556 ± 83^*^	481 ± 104^*^	416 ± 80	370 ± 73
**Anterior hypothalamic area (-1.06)**	156 ± 34	209 ± 31	208 ± 27	284 ± 27	304 ± 28^*a^	299 ± 58^*a^	278 ± 49^*a^	256 ± 32^*a^
**Peduncular part of lateral hypothalamus (−1.06)**	143 ± 28	152 ± 22	180 ± 30	233 ± 18	197 ± 24	197 ± 34	181 ± 29	234 ± 31
**Dorsomedial hypothalamus (-1.46)**	370 ± 54	325 ± 64	494 ± 66	395 ± 40	477 ± 105	357 ± 79	369 ± 47	323 ± 51
**Ventromedial hypothalamus, ventrolateral (−1.46)**	230 ± 62	251 ± 40	159 ± 28	145 ± 31	389 ± 34^*a^	190 ± 58^*a^	300 ± 58^*a^	277 ± 78^*a^
**Ventromedial hypothalamus, dorsomedial (−1.46)**	163 ± 55	232 ± 44	204 ± 38	147 ± 32	264 ± 76^*a^	221 ± 76^*a^	335 ± 90^*a^	420 ± 97^*a^
**Ventromedial hypothalamus, central (−1.46)**	155 ± 72	203 ± 63	210 ± 26	207 ± 20	178 ± 40	98 ± 30	351 ± 128^###*^	406 ± 67^###*^
**Ventrolateral preoptic hypothalamus (+ 0.02)**	237 ± 31	237 ± 73	338 ± 31^#^	382 ± 64^#^	402 ± 68^**^	392 ± 59^**^	425 ± 72	246 ± 48
**Ventromedial preoptic hypothalamus (+ 0.02)**	236 ± 46	309 ± 78	287 ± 81	300 ± 83	416 ± 90^*a^	386 ± 80^*a^	474 ± 112^*a^	418 ± 69^*a^
**Medial preoptic hypothalamus (+ 0.02)**	129 ± 31	160 ± 27	221 ± 43	211 ± 24	326 ± 49^##***^	306 ± 63^##***^	215 ± 39	173 ± 21
**Lateral preoptic hypothalamus (+ 0.02)**	212 ± 8	215 ± 22	300 ± 37^##**^	291 ± 22^##**^	234 ± 32	264 ± 45	233 ± 36	195 ± 30
**Striatum**								
**Nucleus accumbens, core (+ 1.10)**	112 ± 31	81 ± 16	186 ± 47^#a^	182 ± 64^#a^	113 ± 31	106 ± 24	95 ± 19^#a^	174 ± 28^#a^
**Nucleus accumbens, shell (+ 1.10)**	95 ± 21	72 ± 10	159 ± 11	254 ± 66^###$$^	138 ± 26	137 ± 26	130 ± 19	216 ± 29^###$$^
**Caudate putamen (+ 1.10)**	77 ± 9	53 ± 13	55 ± 17	74 ± 23	54 ± 13	35 ± 10	66 ± 15	36 ± 7
**Amygdaloid regions**								
**Medial amygdala, anteroventral (− 1.22)**	111 ± 19	178 ± 15	302 ± 50^###*^	396 ± 76^###*^	301 ± 55	182 ± 31	246 ± 68	230 ± 26
**Medial amygdala, anterodorsal (− 1.22)**	173 ± 30	147 ± 38	372 ± 55^###^	391 ± 84^###^	291 ± 46	215 ± 31	300 ± 47	303 ± 49
**Anterior cortical amygdala area (−1.22)**	151 ± 23	207 ± 52	336 ± 39^##@^	322 ± 62^##^	380 ± 73^*@(^	203 ± 29^*^	214 ± 49	244 ± 44
**Posterolateral cortical amygdala area (−1.22)**	155 ± 36	80 ± 26	373 ± 95^###**^	308 ± 26^###**^	279 ± 67^*^	159 ± 33^*^	188 ± 42	200 ± 40
**Piriform cortex (−1.22)**	107 ± 29	105 ± 28	189 ± 29^#*^	192 ± 44^#*^	166 ± 38	87 ± 11	99 ± 18	132 ± 32
**Basomedial amygdala (−1.22)**	132 ± 30	165 ± 27	357 ± 48^###**^	363 ± 38^###**^	252 ± 47	204 ± 54	247 ± 32	246 ± 51
**Dorsal endopiriform claustrum (−1.22)**	117 ± 32	106 ± 44^$a^	108 ± 13	245 ± 48^$a^	108 ± 36	140 ± 28^$a^	78 ± 25	130 ± 20^$a^
**Ventral endopiriform (−1.22)**	44 ± 21	112 ± 23	149 ± 39^#a^	191 ± 63^#a^	82 ± 35	67 ± 17	99 ± 33^#a^	87 ± 38^#a^
**Central amygdala, capsular (− 1.22)**	120 ± 32	141 ± 49	499 ± 89^###a^	569 ± 74^###a^	206 ± 58	296 ± 45	501 ± 107^###a^	470 ± 54^###a^
**Central amygdala, lateral (−1.22)**	66 ± 24	85 ± 37	460 ± 113^###^	649 ± 88^###^	132 ± 30^*^	293 ± 63^*^	399 ± 72^###^	549 ± 44^###^
**Central amygdala, medial (−1.22)**	57 ± 0.1	67 ± 17	361 ± 66^###^	336 ± 64^###^	145 ± 23	186 ± 55	237 ± 71^#^	314 ± 59^#^
**Lateral amygdala, dorsolateral (−1.82)**	204 ± 34	125 ± 23	173 ± 29^#*^	298 ± 46^#*++^^^	183 ± 43	261 ± 33^+^^	170 ± 29	159 ± 31
**Lateral amygdala, ventrolateral (−1.82)**	181 ± 37	129 ± 26	226 ± 14^##**^	279 ± 48^##**^	226 ± 36^#*^	246 ± 22^#*^	185 ± 25	157 ± 23
**Lateral amygdala, ventromedial (− 1.82)**	213 ± 62	110 ± 21	202 ± 29	216 ± 35	191 ± 26^*^	267 ± 13^*+^^^	187 ± 28	146 ± 19
**Basolateral amygdala, posterior (−1.82)**	100 ± 28	89 ± 20	186 ± 28^###a^	227 ± 25^###a^	140 ± 19	163 ± 28	184 ± 29^###a^	214 ± 20^###a^
**Basolateral amygdala, anterior (−1.82)**	202 ± 33	142 ± 24	292 ± 31^##^	312 ± 49^##^	270 ± 41^#***^	343 ± 43^#***^	226 ± 27	238 ± 33
**Basomedial amygdala, posterior (−1.82)**	173 ± 30	99 ± 25	230 ± 31^##*^	285 ± 33^##*^	226 ± 44^*^	211 ± 36^*^	173 ± 25	183 ± 30
**Bed nucleus of stria terminalis**								
**BNST, medial, ventral (+ 0.02)**	182 ± 21	184 ± 29	376 ± 46^###*^	382 ± 54^###*^	271 ± 47	262 ± 31	274 ± 37	305 ± 59
**BNST, lateral, ventral (+ 0.02)**	163 ± 19	181 ± 28	399 ± 45^###*^	444 ± 51^###*^	218 ± 31	293 ± 37	290 ± 48	348 ± 67
**BNST, lateral, posterior (+ 0.02)**	126 ± 25	149 ± 26	342 ± 29^###^	319 ± 36^###^	253 ± 34^*^	177 ± 13^*^	245 ± 25	290 ± 48
**BNST, lateral, dorsal (+ 0.02)**	144 ± 56	147 ± 35	316 ± 44^###^	394 ± 60^###^	237 ± 36	205 ± 65	272 ± 34	301 ± 50
**BNST, medial, anteromedial (+ 0.02)**	187 ± 31	226 ± 33	345 ± 40^#^	296 ± 42^#^	320 ± 66^#**^	407 ± 104^#**^	226 ± 35	273 ± 60
**Thalamus**								
**Medial habenula (−1.70)**	59 ± 21	163 ± 64	119 ± 33	182 ± 51	128 ± 28	124 ± 24	103 ± 38	101 ± 17
**Lateral habenula (−1.70)**	41 ± 14	81 ± 38	142 ± 33^###^	188 ± 25^###^	156 ± 31^* $$^	113 ± 8^*^	177 ± 37^$$^	127 ± 8
**Paraventricular thalamus (−1.70)**	546 ± 54	577 ± 45	770 ± 73^###a^	832 ± 54^###a^	663 ± 96^*a^	789 ± 75^*a^	814 ± 82^###a *a^	907 ± 92^###a *a^
**Dorsal raphe**								
**Dorsal raphe, dorsal (−4.42)**	134 ± 17	69 ± 18	144 ± 34	257 ± 71^^+^	122 ± 41	167 ± 17	175 ± 59	114 ± 22
**Dorsal raphe, ventral (−4.42)**	71 ± 15	138 ± 63	120 ± 17	161 ± 37	132 ± 72	103 ± 33	137 ± 30	86 ± 20
**Dorsal raphe, intrafascular (−4.78)**	60 ± 23	102 ± 27	145 ± 28	143 ± 26	78 ± 18	161 ± 24	106 ± 26	179 ± 41
**Dorsal raphe, lateral (−4.78)**	115 ± 22	182 ± 45	166 ± 63	159 ± 52	146 ± 35	176 ± 40	183 ± 34	171 ± 35
**Dorsal raphe, caudal (−5.14)**	100 ± 27	87 ± 23	170 ± 58	135 ± 39	85 ± 29	68 ± 27	139 ± 41	116 ± 33

Values represent number of Fos-positive cells/mm.^2^. #*p* < 0.05; ##*p* < 0.01; ###*p* < 0.001: two-way interaction effect naive deferiprone vs. naive vehicle or PST deferiprone vs PST vehicle groups; $*p* < 0.05; $$*p* < 0.01: two-way interaction effect WT vehicle vs. KO vehicle or WT deferiprone vs KO deferiprone groups; *p < 0.05; **p < 0.01; ***p < 0.001: two-way interaction effect WT naive vs. WT PST or KO naive vs. KO PST groups; + *p* < 0.05; +  + *p* < 0.01: three-way interaction effect KO naive vehicle vs. KO naive deferiprone or KO PST vehicle vs. KO PST deferiprone groups; †*p* < 0.05: three-way interaction effect WT PST deferiprone vs. KO PST deferiprone groups; @*p* < 0.05: three-way interaction effect WT naive vehicle vs. WT PST vehicle or WT naive deferiprone vs. WT PST deferiprone groups; ^*p* < 0.05; ^^*p* < 0.01: KO naive vehicle vs. KO PST vehicle or KO naive deferiprone vs. KO PST deferiprone groups; (*p* < 0.05 WT PST vehicle vs. WT PST deferiprone groups; a = overall effect found in all groups and no interaction effect. Labelled group represents increased c-Fos expression against comparison group. Values are presented as the mean ± SEM, *n* = 5–9 per group

WT = wild-type, KO = 5-HTT knock-out, BNST = bed nucleus of stria terminalis, PST = Porsolt swim test

In subnuclei of the lateral amygdala, a significant stress × genotype × treatment interaction was observed (*F*_(1,50)_ = 9.14, *p* < 0.01) with the post hoc test revealing that in behaviourally naive 5-HTT KO mice, deferiprone resulted in increased c-Fos expression compared to vehicle treatment (*p* < 0.01). In addition, in the 5-HTT KO mice treated with vehicle, swim stress exposure increased c-Fos expression (*p* < 0.01). Meanwhile, in swim stress–exposed mice, 5-HTT KO mice treated with deferiprone had decreased c-Fos expression compared to vehicle treatment (*p* < 0.05) (Fig. 3a). In subnuclei of the dorsal raphe, a significant stress × genotype × treatment interaction was observed (*F*_(1,47)_ = 6.24, *p* < 0.05) with the post hoc test revealing that in behaviourally naive mice, 5-HTT KO mice treated with deferiprone had increased c-Fos expression compared to vehicle-treated mice (*p* < 0.05) (Fig. 3b). In subnuclei of the lateral septum, a trend for a stress × genotype × treatment interaction was observed (*F*_(1,45)_ = 3.50, *p* = 0.068) with the post hoc test revealing that in swim stress–exposed mice, the WT mice treated with deferiprone had greater c-Fos expression compared to 5-HTT KO mice treated with deferiprone (*p* < 0.05) (Fig. 3c).

### Functional Connectivity Network and Hubs Identify Key Regions of Activity Following Swim Stress and Deferiprone Treatment in WT and 5-HTT KO Mice

**Fig. 4:**
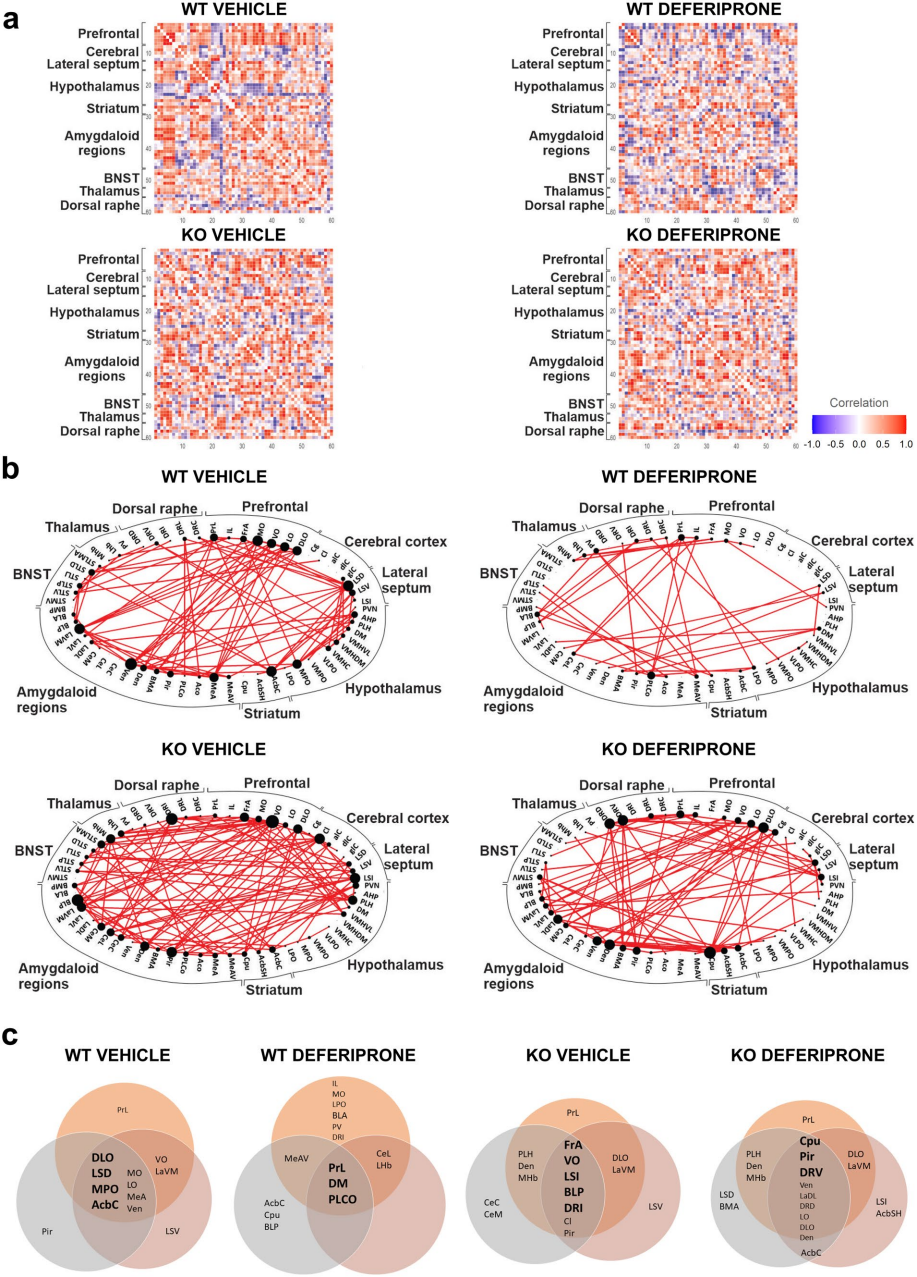
Functional connectivity network and hub regions following deferiprone treatment in WT and 5-HTT KO mice. **a** Connectivity matrix for inter-regional correlation following deferiprone treatment in both WT and 5-HTT KO mice was created using Pearson’s correlation. **b** The functional connectivity network in each group was created using the strongest (Pearson’s *r* ≥ 0.83) correlation between regions. The size of nodes is proportional to the number of significant inter-regional correlation while the lines depict the edge between two nodes. **c** Hubs for the functional connectome in WT and KO mice treated with deferiprone. Hubs are central regions of information integration, and were identified in bold by determining if they were in the 80th percentile for number of nodes in the low (grey) *r* ≥ 0.78, primary (orange) *r* ≥ 0.83 and high (red) *r* ≥ 0.87 correlation network as well as the 80th percentile for betweenness centrality. WT = wild-type; KO = knock-out; BNST = bed nucleus of the stria terminalis

**Fig. 5:**
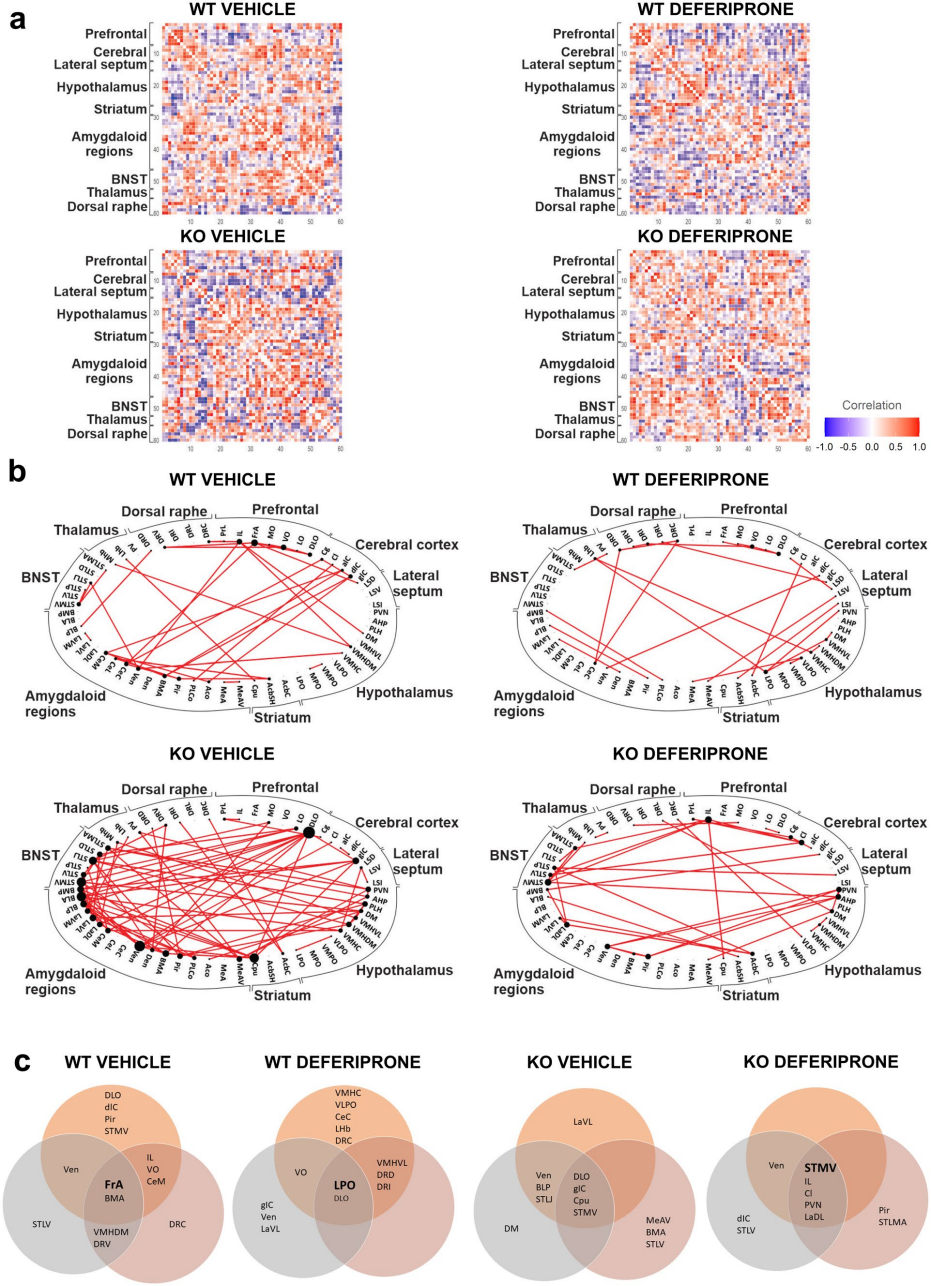
Functional connectivity network and hub regions following deferiprone treatment and swim stress in WT and 5-HTT KO mice. **a** Connectivity matrix for inter-regional correlation following deferiprone treatment and swim stress in both WT and 5-HTT KO mice was created using Pearson’s correlation. **b** The functional connectivity network in each group was created using the strongest (Pearson’s *r* ≥ 0.83) correlation between regions. The size of nodes is proportional to the number of significant inter-regional correlation while the lines depict the edge between two nodes. **c** Hubs for the functional connectome in WT and KO mice exposed to swim stress and deferiprone treatment. Hubs are central regions of information integration, and were identified in bold by determining if they were in the 80th percentile for number of nodes in the low (grey) *r* ≥ 0.78, primary (orange) *r* ≥ 0.83 and high (red) *r* ≥ 0.87 correlation network as well as the 80th percentile for betweenness centrality. WT = wild-type; KO = knock-out; BNST = bed nucleus of the stria terminalis; PST = Porsolt swim test

**Fig. 6:**
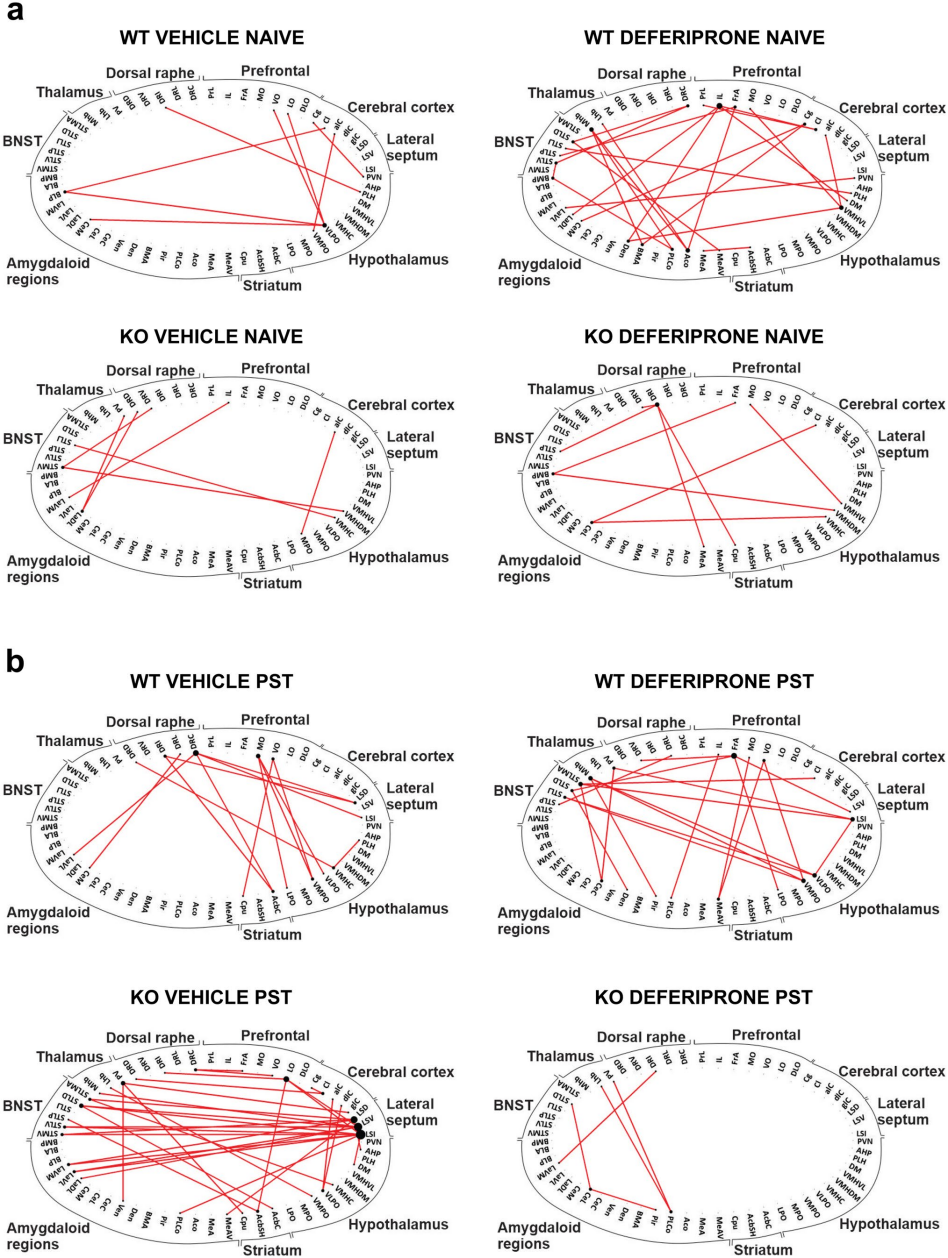
Functional negative correlation network following deferiprone treatment and swim-stress exposure in WT controls and 5-HTT KO mice. The functional negative correlation network was characterised in behaviourally naive (**a**) and following swim-stress exposure (**b**)**.** The negative correlation network for each group was created using the strongest negative (Pearson’s *r* ≤  −0.87) between regions. The size of nodes is proportional to the number of significant inter-regional negative correlation while the lines depict the edge between two nodes

The next analyses aimed to characterise the network-wide functional connectome following deferiprone treatment in behaviourally naive (Fig. 4) and swim stress/PST exposed (Fig. 5) mice. Increased inter-regional correlation suggests that regions are acting together while negative correlation (Fig. 6) suggests modulation via inhibitory networks.

Graph theory–based analyses on c-Fos expression revealed unique inter-regional correlation matrices following deferiprone in behaviourally naive mice (Fig. 4a) and following swim-stress exposure (Fig. 5a) in both genotypes. The positive correlation functional network revealed that in behaviourally naive mice, there was a greater number of inter-regional correlations in WT mice treated with deferiprone compared to 5-HTT KO mice treated with deferiprone (Fig. 4b). In comparison, not many significant connections were observed in the negative correlation functional network (Fig. 6a). When analysing for hubs, WT deferiprone-treated mice had hubs localised to the prelimbic cortex, dorsomedial hypothalamus and the posterolateral cortical amygdala; whereas 5-HTT KO deferiprone-treated mice had hubs mainly localised to the dorsal raphe, caudate putamen and piriform cortex (Fig. 4c).

The functional network in stress–exposed mice revealed highly connected nodes in the 5-HTT KO vehicle-treated group, including regions of the amygdala, striatum and BNST (Fig. 5b). On the other hand, there were fewer inter-regional correlations in the deferiprone-treated groups in either genotype. The negative correlation network revealed that in 5-HTT KO mice following swim-stress exposure and vehicle treatment, the lateral septum was a highly connected node with preferential connectivity to the lateral amygdala and BNST (Fig. 6b). Brain regions which were hubs following swim-stress exposure and deferiprone treatment were the lateral preoptic hypothalamus in WT mice and the BNST in 5-HTT KO mice (Fig. 5c). The hubs were informed by the degree and betweenness centrality for each region (Figs. S3 and S4). Degree represents number of significant inter-regional correlations for the region while betweenness centrality is the fraction of all shortest paths in the network which pass through the region [50].

### Swim Stress Results in an Increase in Modular Structuring of the Brain in WT But Not in 5-HTT KO Mice

**Fig. 7:**
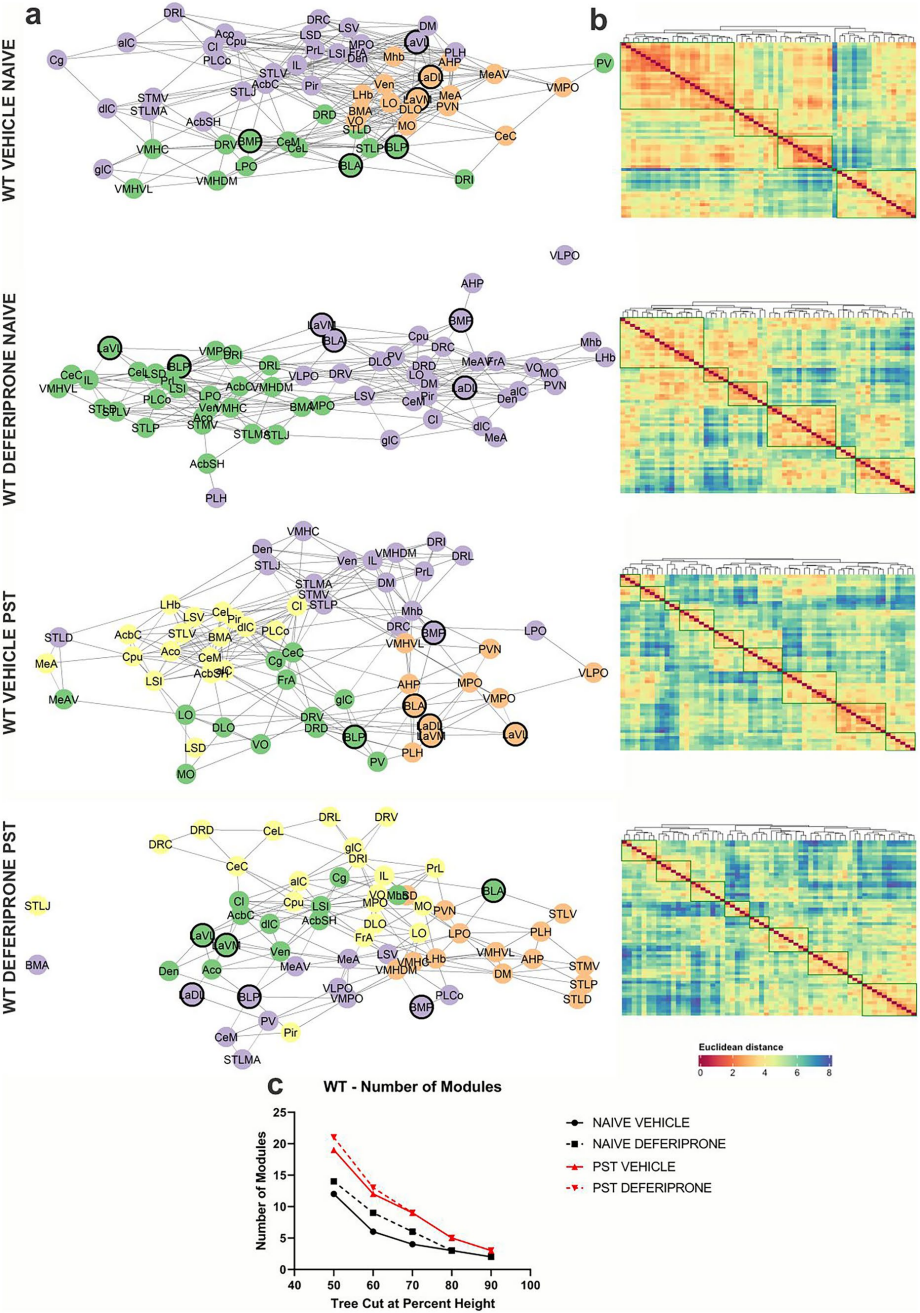
Community detection and hierarchical clustering on behaviourally naive and swim stress–exposed WT mice. **a** Graph-theory based community detection analysis of the functional network following deferiprone treatment and swim-stress exposure. An arbitrary Pearson’s *r* > 0.6 was used for visualisation purposes. Different colours around each node represent respective modules of activity. Regions in bold include the lateral, basolateral and basomedial amygdala. **b** Hierarchical clustering of the Euclidean distance following deferiprone treatment and swim stress exposure. Dendrogram was cut at 70% height for clustering visualisation. **c** Number of modules of each treatment group at various tree cuts of the hierarchical clustering dendrogram. WT = wild-type

**Fig. 8:**
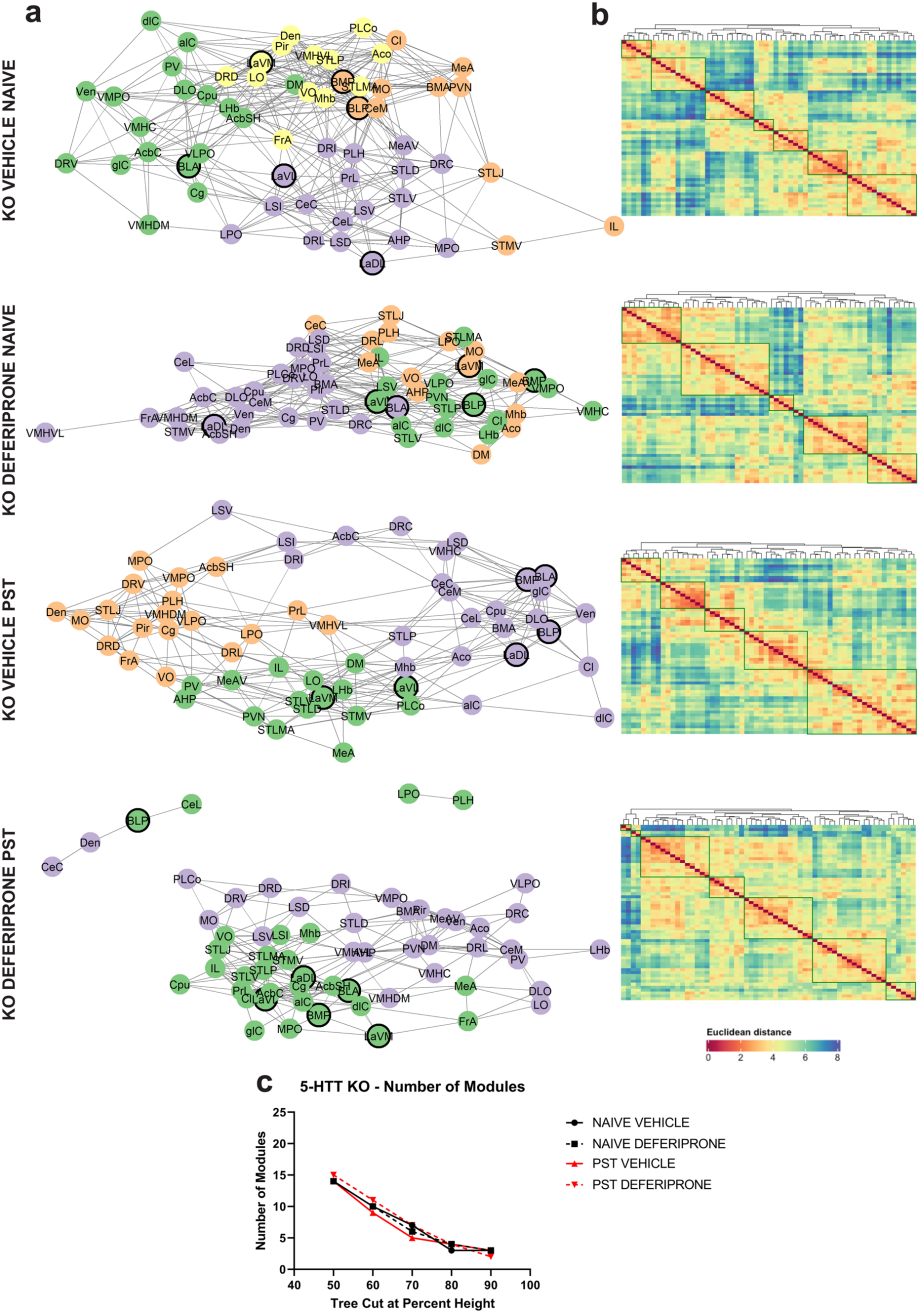
Community detection and hierarchical clustering on behaviourally naive and stress–exposed 5-HTT KO mice. **a** Graph-theory based community detection analysis of the functional network following deferiprone treatment and swim-stress exposure. An arbitrary Pearson’s *r* > 0.6 was used for visualisation purposes. Different colours around each node represent respective modules of activity. Regions in bold include the lateral, basolateral and basomedial amygdala. **b** Hierarchical clustering of the Euclidean distance following deferiprone treatment and swim-stress exposure. Dendrogram was cut at 70% height for clustering visualisation. **c** Number of modules of each treatment group at various tree cuts of the hierarchical clustering dendrogram. KO = knock-out

To determine changes in network modular organisation of swim stress/PST exposure as well as deferiprone treatment in WT (Fig. 7) and 5-HTT KO (Fig. 8) mice, graph theory–based community detection and hierarchical clustering of c-Fos expression correlation dataset was conducted. In addition, the modular organisation of the community detection network can be utilised to determine the clustering of important regions following deferiprone treatment.

Community detection analysis revealed an increase in the number of modules and organisation of the network following swim-stress exposure in WT mice (Fig. 7a). This effect was not observed in 5-HTT KO mice where the number of modules did not increase following swim stress (Fig. 8a). In further confirmation of the increased modularity of WT mice following swim-stress exposure, hierarchical clustering of the Euclidean distance resulted in an increase in number of modules in WT (Fig. 7b) which was not observed in 5-HTT KO (Fig. 8b) mice. To ensure that tree cutting of the dendrogram at different thresholds was not responsible for the observed increase in modularity of WT mice, the number of modules at progressively increasing tree cut heights was characterised. At a range of tree cuts of the dendrogram of the hierarchical clustering dataset, WT mice exposed to swim stress maintained the increase in modularity compared to behaviourally naive controls (Fig. 7c). In comparison, tree cutting at different dendrogram heights had no effect on the number of modules in 5-HTT KO mice (Fig. 8c). Furthermore, regardless of the genotype, deferiprone had no effect on the number of modules in either modular clustering measure.

When looking at regions which had been previously shown to be affected by deferiprone treatment and swim-stress exposure in the 5-HTT KO mice, the lateral, basolateral and basomedial amygdala had changes to c-Fos expression (Table 1). Therefore, their properties in the community detection network were examined. When looking at each group separately (Figs. 7a and 8a), we found that these regions were distributed across the modules and the network in all groups except the KO deferiprone PST group, in which these regions were highly clustered, and all found within the same module.

## Discussion

### Antidepressant-Like Effects of Deferiprone

Our study is the first to examine the effects of acute deferiprone treatment on depression-related behaviours. We provide the first evidence of antidepressant-like effects of acute deferiprone, as immobility time of 5-HTT KO mice in the Porsolt swim test (PST) was reduced 1 hour after deferiprone administration. The 5-HTT KO mice have been shown to have increased immobility time in the PST, indicative of a depression-like phenotype, which is consistent with the observations in our study [18, 24]. Ruling out any possible confounding effects on locomotor activity, we found that deferiprone had no effect on locomotion in 5-HTT KO mice 1 hour post-injection. We also revealed that deferiprone reduced latency to feed in the novelty-suppressed feeding test (NSFT) in both genotypes. Altogether, these findings suggest that deferiprone has antidepressant-like properties [24, 53–55].

The antidepressant-like effects of deferiprone are unlikely to involve the 5-HT transporter, as these behavioural effects (revealed in both the PST and NSFT) were found in 5-HTT KO mice, which completely lack the 5-HTT protein, the primary target of SSRIs [24]. Acute treatments with glutamatergic antidepressants, such as ketamine, have been shown to exert sustained antidepressant-like effects in the PST [53, 56, 57]. However, we did not find such effect of deferiprone when assessed 24 hours post-injection in the PST.

Further supporting the antidepressant-like actions of deferiprone, we showed that acute deferiprone reduced latency to feed in the NSFT in both genotypes. Notably, antidepressant-like effects of classic monoaminergic antidepressants in the NSFT are typically observed after chronic but not acute administration [54, 58]. In our NSFT experiment, deferiprone had actions similar to fast-acting antidepressants, such as ketamine, which act primarily on the glutamatergic system and have also been shown to reduce latency to feed 1 hour post-treatment [45, 55, 57]. While there are also previous reports of 5-HTT KO mice having reduced latency to feed in the NSFT [59, 60], we did not reveal any difference between genotypes in our study. This may be due to the acute stressor in the form of the intraperitoneal injection 1 hour before testing, which was not present in prior studies, or due to background strain differences [59, 60]. We observed that mice which were to receive deferiprone treatment had slightly greater weight loss during the 24-hours fasting period (Fig. S2a) which potentially confounded their desire for food consumption during the NSFT protocol. However, there was only a few-percent difference in terms of weight loss between treatment groups, and vehicle-treated mice also lost substantial body weight indicating they were sufficiently hungry to perform the test. Furthermore, deferiprone treatment conversely reduced pellet consumption selectively in 5-HTT KO mice in their home cage following the NSFT protocol and had no effect on WT controls (Fig. S2b). This further confirms that the reduction in latency to feed in the NSFT induced by deferiprone was not due to an increased desire for food consumption.

In the post-NSFT period, vehicle-treated 5-HTT KO mice had increased food pellet consumption compared to WT controls when placed back in their home cages (Fig. S2b). Another study has found that 5-HTT KO mice have no differences in their home-cage pellet consumption following the NSFT protocol [59]; however, this discrepancy may be explained by strain differences. There is evidence to indicate that C57Bl/6J 5-HTT KO mice do have increase home-cage food consumption [61, 62] and human ‘s’ allele carriers of the 5-HTTLPR region of the gene have increased emotional state associated food consumption [63] which may underpin our observations.

Since 5-HTT KO mice are also considered a model of anxiety [64] and the NSFT is relevant to anxiety-like behaviours as well as being sensitive to acute anxiolytic drugs [65, 66], we assessed the effect of acute deferiprone in another anxiety-like behavioural paradigm, the light–dark box test. We were able to confirm that 5-HTT KO mice spend less time in the light compartment when compared to WT animals. We also revealed an anxiogenic-like effect of acute deferiprone in both genotypes. In a mouse model of tauopathy, Rao et al. [67] found that chronic deferiprone treatment had an anxiolytic-like effect. However, this was in a model of tauopathy following 16 weeks of treatment and using time spent in the outer arena zone as a measure of anxiety-like behaviours, which was substantially different from our acute study. While our results in the light–dark box test are in contrast to our antidepressant/anxiolytic-like effect of deferiprone we found in the NSFT, there is evidence that iron deficiency anaemia is linked to increased prevalence of anxiety disorders [68]. There is also data suggesting that prolonged iron deficiency results in anxiety-like behaviours in rodents [69]. Notably, deferiprone also reduced total distance travelled in the light–dark box test, which could potentially confound the overall interpretation on anxiety-like phenotype. Interestingly, our effect of deferiprone on anxiety level is similar to the anxiogenic-like effect previously reported following acute treatment with SSRIs [70–73]. Notably, only 5-HTT KO mice had reduced number of bouts to the light compartment following acute deferiprone treatment indicating a further genotype specific effect of deferiprone in a model of serotonergic dysfunction. In summary, these results indicate that deferiprone’s therapeutic potential is limited to an antidepressant-like effect.

We also found that deferiprone increased locomotor activity in both genotypes in the first 40 minutes of the post-injection period. Critically, there was no effect on locomotion at the 60-minutes post-injection timepoint, which indicates that the drug-induced effects on locomotor activity are not confounding our observation in the PST.

### Acute Deferiprone Treatment Does Not Have an Effect on Metal Levels

To determine whether the acute antidepressant-like effects of deferiprone were due to alteration of iron levels, levels of several metals were measured in various brain regions and in the blood following acute deferiprone treatment. We found that deferiprone did not change total iron levels in the prefrontal cortex, striatum, brainstem or peripheral blood in either genotype, indicating that the acute antidepressant-like effects may be mediated via an iron-independent mechanism or by changes in intracellular iron pools. Indeed, the fact that deferiprone did not reduce total iron levels in any brain region of interest may reflect an iron redistribution effect of the drug rather than iron removal [74].

In comparison, there is evidence that chronic deferiprone treatment reduces iron levels in the hippocampus in WT rats; however, the study failed to disclose the dosage of deferiprone administration and may be much higher than our study [75]. Furthermore, Mehrpouya et al. [75] found that chronic deferiprone treatment increased immobility time in the PST, which was linked to lower levels of iron. It has been previously documented that iron deficiency results in depression-associated symptoms in both humans and animal models [76, 77]. Our results indicate that there is an iron level and behavioural difference when comparing acute and chronic delivery of deferiprone which may be acting via different biological mechanisms. Deferiprone has also been shown to chelate other metals in a less selective manner [78]; however, we found that acute deferiprone treatment had no effect on other metals of interest in brain regions and circulating blood. Finally, it is still possible that deferiprone would have perturbed the labile iron pool, which is only 2% of the total tissue iron and would not have been detectable in a study of this power. Notably, this labile pool is the one that interacts with tyrosine and tryptophan hydroxylases.

In terms of the effect on metal levels due to the genetic ablation of the 5-HTT, we found no differences of any metals at any brain region of interest. These results are in line with a previous report of unchanged iron level in the hippocampus in another 5-HTT KO mouse, although that same study found reduced iron in the midbrain [79]. Notably, the midbrain is only one section of the brainstem and therefore iron level dysregulation may occur selectively in subregions of the brainstem. The fact that we found no genotype effect on iron levels in the prefrontal cortex, striatum and the brainstem would indicate that there is not a general iron alteration across many regions of the 5-HTT KO mouse model. However, in line with the altered metals levels hypothesis, we interestingly found increased levels of iron and zinc in the blood of 5-HTT KO mice. Although there is evidence for depression severity being correlated with elevated levels of iron in various brain regions [8], as well as thalassemia patients with depression having elevated levels of the iron storage protein transferrin [9], our results would indicate that acute deferiprone is acting in via iron-independent mechanism in a mouse model of depression without iron dysregulation in the brain. While we did not reveal any effect of deferiprone on blood metals levels, it would be interesting to determine whether acute deferiprone is having an effect on iron and zinc transporter or storage, which may be more relevant to the rapid actions of the drug instead of total levels of each metal. Also, it is worth noting that it is still possible that deferiprone and/or 5-HTT mutation could have perturbed the labile iron pool, which is only 2% of the total tissue iron and would not have been detectable in a study of this power.

### Brain Region Activity Following Swim Stress and Deferiprone

Using c-Fos expression immunostaining, we then determined the relevant brain regions activated by deferiprone in animals exposed to PST as well as in mice naive from any behavioural testing. Indeed, there is evidence that immobility time in the PST can be used as a proxy for stress-coping behaviour, which has been shown to be aberrant in MDD and 5-HTT KO mice [18, 80]. Our data show that, in response to swim stress, subdivisions of the amygdala were differentially activated in 5-HTT KO compared to WT mice, potentially reflecting the increased immobility behaviour and/or aberrant stress response in 5-HTT KO mice. While altered stress responses have been previously revealed in both 5-HTTLPR s allele carrier MDD patients and 5-HTT KO mice [22, 81], our c-Fos study is the first to look at neuronal activity following swim stress in 5-HTT KO mice. Notably, the amygdala has been implicated in the stress response and processing of threatening stimuli in both humans and rodents [82, 83]. We observed that there was increased c-Fos expression following swim stress exposure in subnuclei of the lateral amygdala of 5-HTT KO mice while, comparatively, there was a WT-selective increase in c-Fos expression in cortical amygdala regions. In line with that, s allele carriers of the 5-HTTLPR have been shown to have increased basolateral amygdala activity following an exposure to a psychological stressor [82, 84]. Prior research also suggests that the lateral amygdala is involved in mediating adverse environmental exposures [85]. Therefore, it is possible that subdivisions of the amygdala are over-activated in response to an acute environmental stressor in the 5-HTT KO mice.

Deferiprone also increased c-Fos expression in several stress-related brain regions in animals of both genotypes in behaviourally naive mice, which could underlie the antidepressant-like effects observed in both the PST and NSFT. Most of these regions have been shown to have increased c-Fos expression following monoaminergic and glutamatergic antidepressant treatment [30–35, 86, 87]. We found a 5-HTT KO-selective increase in c-Fos expression following deferiprone in subnuclei of the lateral amygdala and dorsal raphe. This could be explained by the changes to serotonergic availability in the dorsal raphe, as well as changes to amygdala structure and morphology in the 5-HTT KO mouse [36, 88].

As previously stated, the current findings show that vehicle-treated 5-HTT KO mice had increased c-Fos expression in the lateral amygdala following swim-stress exposure. In contrast, 5-HTT KO mice that were treated with deferiprone prior to swim-stress exposure had a reduction in c-Fos expression in the lateral amygdala, thus reversing the increased neuronal activity of this region following PST exposure. The attenuation of c-Fos expression following existing antidepressant treatment and swim stress in certain brain regions has been attributed to these regions being involved in the therapeutic response of these drugs [35, 82–84, 89, 90]. Monoaminergic antidepressants have not shown an effect on the lateral amygdala following PST and antidepressant exposure but these were done only on WT animals thus far [34, 91]. It is possible that deferiprone is inhibiting the increase in neuronal activity of the lateral amygdala in the 5-HTT KO mice following swim-stress exposure, resulting in reduced immobility time in the PST.

In the current study, the lateral septum was also implicated in the interaction between deferiprone treatment and swim-stress exposure in a genotype-specific manner. We found that following swim-stress exposure, WT mice had increased c-Fos expression in subnuclei of the lateral septum compared to 5-HTT KO mice. Lesions to the lateral septum have been shown to increase immobility time in the PST indicating a link between the function of this region and behavioural response [92]. Furthermore, the SSRI drug paroxetine was shown to attenuate stress–induced increase in c-Fos expression in the lateral septum suggesting a link between antidepressant response and the lateral septum [89]. Therefore, in addition to the lateral amygdala, deferiprone is interacting in a genotype-specific manner to reduce neuronal activity in the lateral septum in the 5-HTT KO mouse model of depression.

### Functional Network Following Swim Stress and Deferiprone

Our functional connectome analyses in behaviourally naive 5-HTT KO mice (i.e. not exposed to swim stress) treated with deferiprone, identified the dorsal raphe and caudate putamen as potential hubs of interest. The functional connectome is a temporal representation of the relationship between anatomical brain regions as defined by shared neuronal activity patterns. Hubs are defined as brain regions which have the greatest information integration within the functional network [48, 50]. Therefore, they are likely to play an important role in the behavioural response to a stimulus as they have extensive co-activity patterns with numerous brain regions. The caudate and prefrontal cortex have been shown to have increased functional connectivity following ketamine treatment in MDD patients and increased brain connectivity of these regions is recognised as a marker of successful antidepressant treatment [40]. Although the prefrontal cortex was not identified as a hub in the KO deferiprone naive group, the orbital cortex was a heavily connected node, with a node being defined as a vertex in the network. Furthermore, in the WT deferiprone naive group, the prelimbic cortex was shown to be a hub, indicating the effect of deferiprone on functional connectivity in the prefrontal cortex. These results are likely to reflect that regions of the prefrontal cortex and the caudate putamen have shared neuronal activity patterns with numerous brain regions, and are therefore likely to play an important role in the behavioural response to deferiprone treatment. Therefore, similar to the findings of Abdallah et al. [40], the antidepressant-like activity of deferiprone may be mediated through an increase in global functional connectivity of the prefrontal cortex and caudate putamen, which needs to be further explored.

Looking at the functional connectome of 5-HTT KO mice exposed to swim stress, the lateral septum was a highly connected node for the negative correlation, or potentially inhibitory network [48]. Notably, its significant connections within the negative correlation network included the lateral amygdala, in line with which the selective increase in c-Fos expression in the 5-HTT KO mice following swim stress. Interestingly, following deferiprone treatment, we did not observe significant connections of the lateral septum in the negative correlation network of the 5-HTT KO mice, suggesting that the drug may alter the aberrant functional connectivity in the 5-HTT KO mice. The lateral septum has an inhibitory role in modulating the HPA axis and exhibits reduced serotonin levels in response to acute stress [92, 93]. It was also not surprising to find projections to the lateral amygdala in the negative correlation network due to altered morphology in this region in 5-HTT KO mice [36]; however, it does provide novel evidence for a potential inhibitory interaction between these regions in response to acute stress. The current study’s findings reinforce the role of the lateral septum in response to swim-stress exposure, and that serotonergic availability in the lateral septum, which is altered in the 5-HTT KO mice, may play a role in the depression-related behaviour.

Our results show that there is increased modularity of the functional network in response to swim stress selectively in WT mice. Network modularity is defined as the parcellation of the overall network into sub-networks which may be responsible for distinctive activity due to shared co-activity patterns [47, 50]. Changes in network modularity have been implicated in depression and response to stressors, as well as other psychiatric disorders [42, 43, 94]. The different effect that an acute stressor has on network properties such as modularity in the 5-HTT KO model of depression needs to be further explored. When looking at regions that deferiprone has been shown to be alter c-Fos expression following PST exposure, the lateral/basolateral/basomedial amygdala were implicated. In the community detection analysis, these regions were distributed in the network and modules of all groups, except for the 5-HTT KO group, where they were shown to be highly clustered, suggesting coactivation of these regions. There is evidence to suggest that regions which are highly clustered are responsible for certain neurophysiological functions [51]. Therefore, it is possible that these regions are acting together, resulting in the antidepressant-like actions of deferiprone to modulate the swim stress.

## Limitations and Conclusions

While our current findings provide evidence for potential antidepressant-like effects of deferiprone, further research is required to determine the molecular and cellular mechanisms mediating its therapeutic actions. Further research must also explore the changes that deferiprone has on the functional network and its relevance to behavioural outcomes. One potential explanation for the antidepressant-like properties of deferiprone may be mediated via brain-derived neurotrophic factor (BDNF) signalling, which is proposed to play a key role in depression and synaptic plasticity [95]. 5-HTT KO mice have reduced expression of BDNF in multiple regions of the brain [96–98] and altered hippocampal long-term potentiation [44]. In addition, deferiprone has been shown to increase levels of BDNF [99]. Therefore, an exploration of acute deferiprone treatment on BDNF signalling and neural plasticity would be an important avenue to explore.

Another possible avenue would be to measure reactive oxygen species as it has been implicated in MDD pathophysiology and influenced by iron dysregulation [11, 13]. In addition, as 5-HTT KO mice also have elevated oxidative stress markers [26], it would be important to explore whether deferiprone is also reducing oxidative stress in 5-HTT KO mice.

The present findings indicate that deferiprone has similarities to both typical antidepressants (e.g. SSRIs) and the new class of fast-acting antidepressants (e.g. ketamine), in both behavioural outcome and brain activity. While our data suggest that the acute antidepressant-like effects of deferiprone is independent of brain iron levels, it is worth noting that our study is underpowered to detect any potential change on the labile iron pool and did not look at other key markers relevant to iron signalling, such as ferritin or transferrin levels. In addition, deferiprone-dependent changes in distribution of iron intracellular pools cannot be ruled out.

In 5-HTT KO mice, deferiprone caused a reduction in the overactivity of the lateral amygdala and lateral septum in response to stress exposure. These findings also provide the first evidence for changes in the functional connectome of the 5-HTT KO mice following swim-stress exposure and the relevance for increased functional brain connectivity of the caudate putamen and the prefrontal cortex in the actions of deferiprone, as well as the lateral septum in the negative correlation network. In conclusion, the current findings indicate that deferiprone is a therapeutic compound with novel antidepressant-like characteristics. Deferiprone appears to mediate its antidepressant-like actions via the lateral amygdala and lateral septum. The behavioural and neural activation profile of deferiprone seems unique to the 5-HTT KO mouse model of depression. While our current findings using the 5-HTT KO mouse model is relevant to SSRI-resistant depression, further studies assessing deferiprone on models of stress-induced depressive-like state in mice such as chronic social defeat stress would be very interesting.

## Supplementary Information

Below is the link to the electronic supplementary material.

Supplementary file1 (JPG 293 KB)

Supplementary file2 (JPG 336 KB)

Supplementary file3 (JPG 235 KB)

Supplementary file4 (JPG 238 KB)

Supplementary file5 (DOCX 818 KB)

Supplementary file6 (PDF 553 KB)

Supplementary file7 (PDF 526 KB)

Supplementary file8 (PDF 535 KB)

Supplementary file9 (PDF 544 KB)

Supplementary file10 (PDF 517 KB)

Supplementary file11 (PDF 526 KB)

Supplementary file12 (PDF 544 KB)

Supplementary file13 (PDF 553 KB)

Supplementary file14 (PDF 535 KB)

Supplementary file15 (PDF 508 KB)

Supplementary file16 (PDF 562 KB)

Supplementary file17 (PDF 508 KB)
