# Supplementary figures and images for: Novel Antidepressant-Like Properties of the Iron Chelator Deferiprone in a Mouse Model of Depression
Source: Neurotherapeutics. 2022 Jul 21;19(5):1662–85. doi: 10.1007/s13311-022-01257-0 (PMC9606181; doi:10.1007/s13311-022-01257-0)

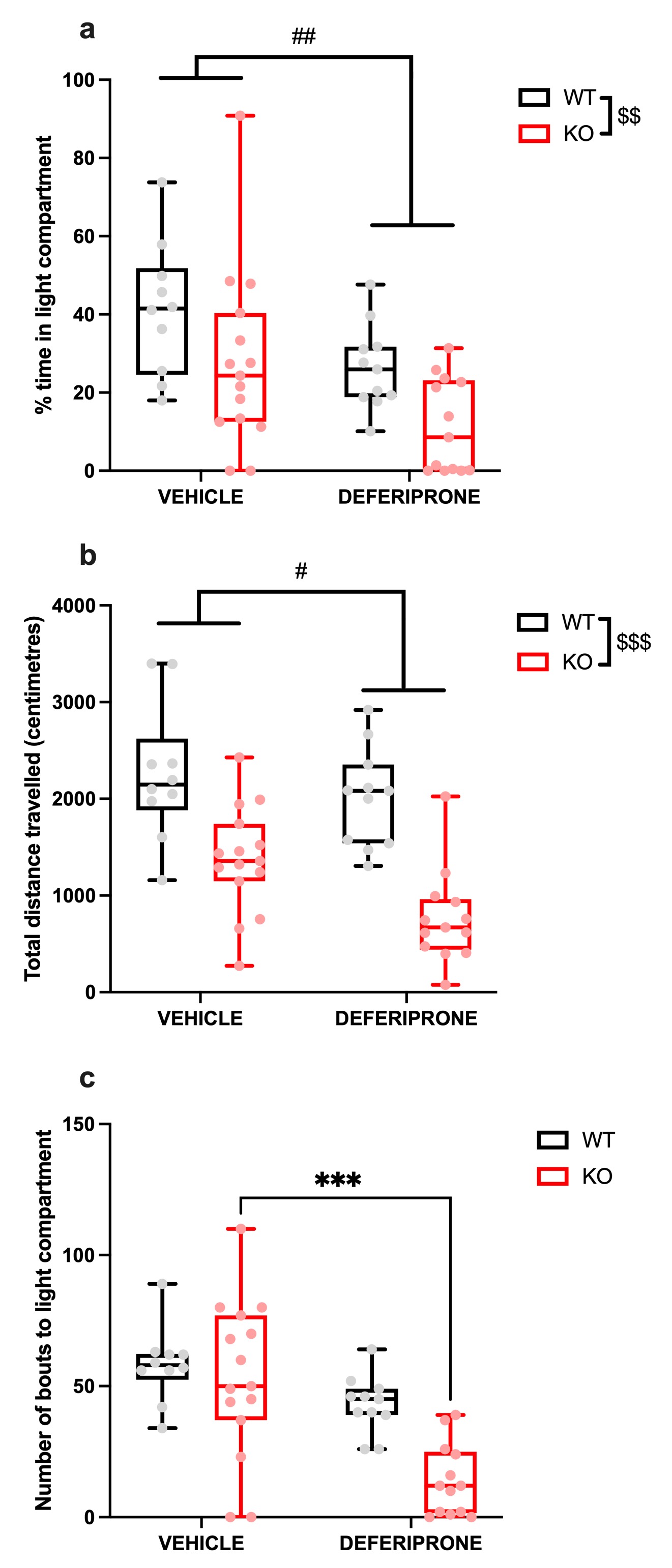

Supplement: Supplementary file 1 — Supplementary file1 (JPG 293 KB) [file 13311_2022_1257_MOESM1_ESM.jpg]

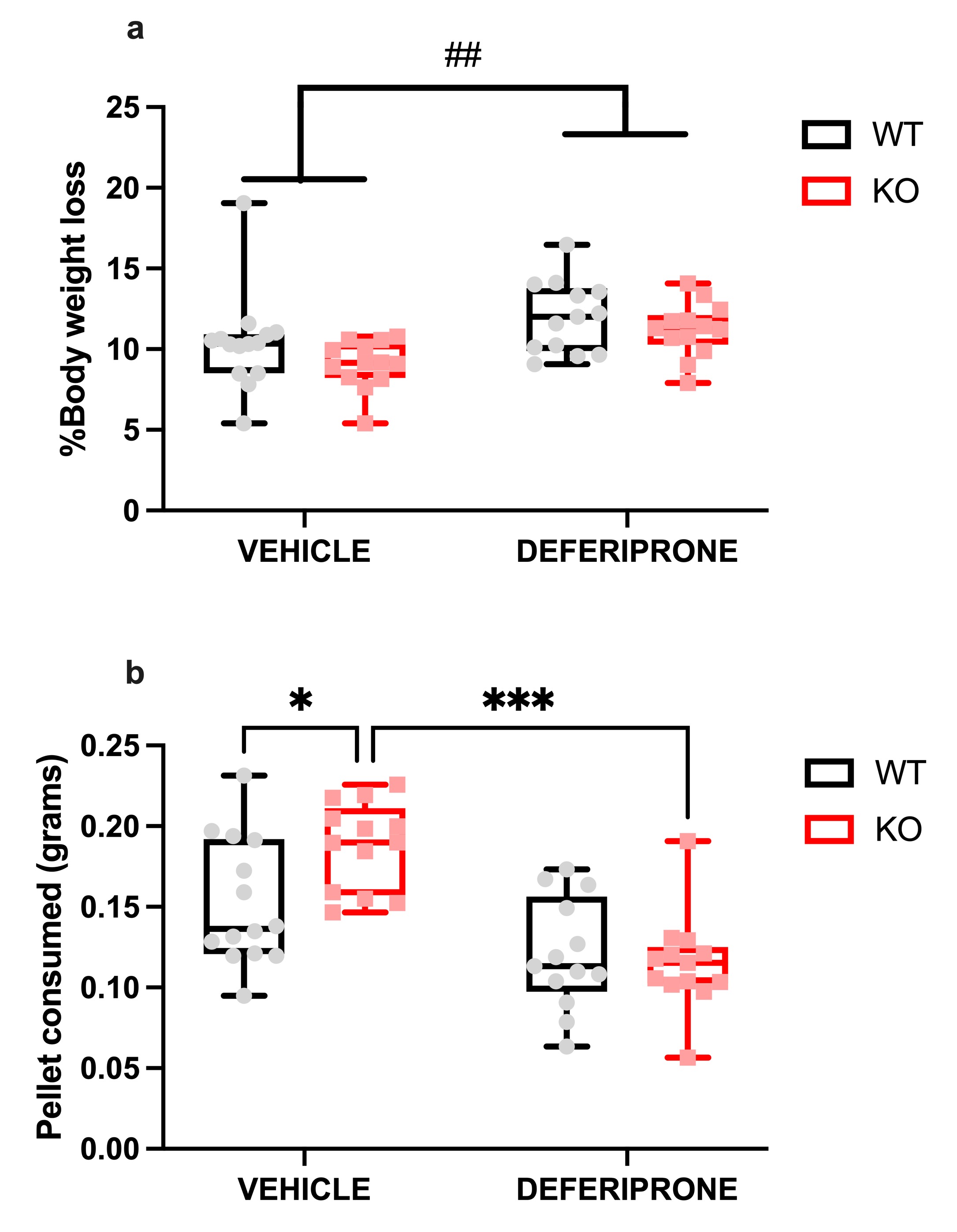

Supplement: Supplementary file 2 — Supplementary file2 (JPG 336 KB) [file 13311_2022_1257_MOESM2_ESM.jpg]

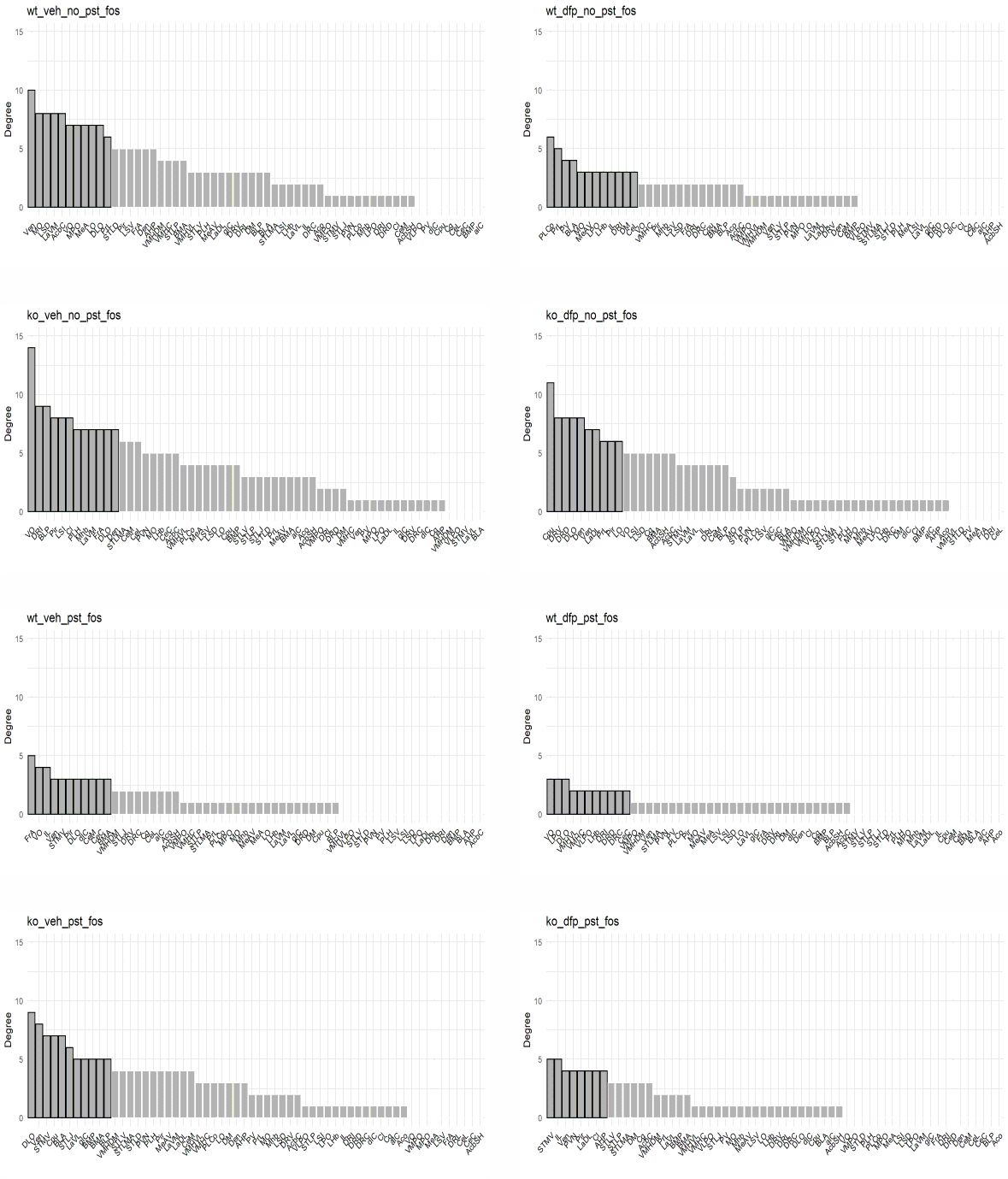

Supplement: Supplementary file 3 — Supplementary file3 (JPG 235 KB) [file 13311_2022_1257_MOESM3_ESM.jpg]

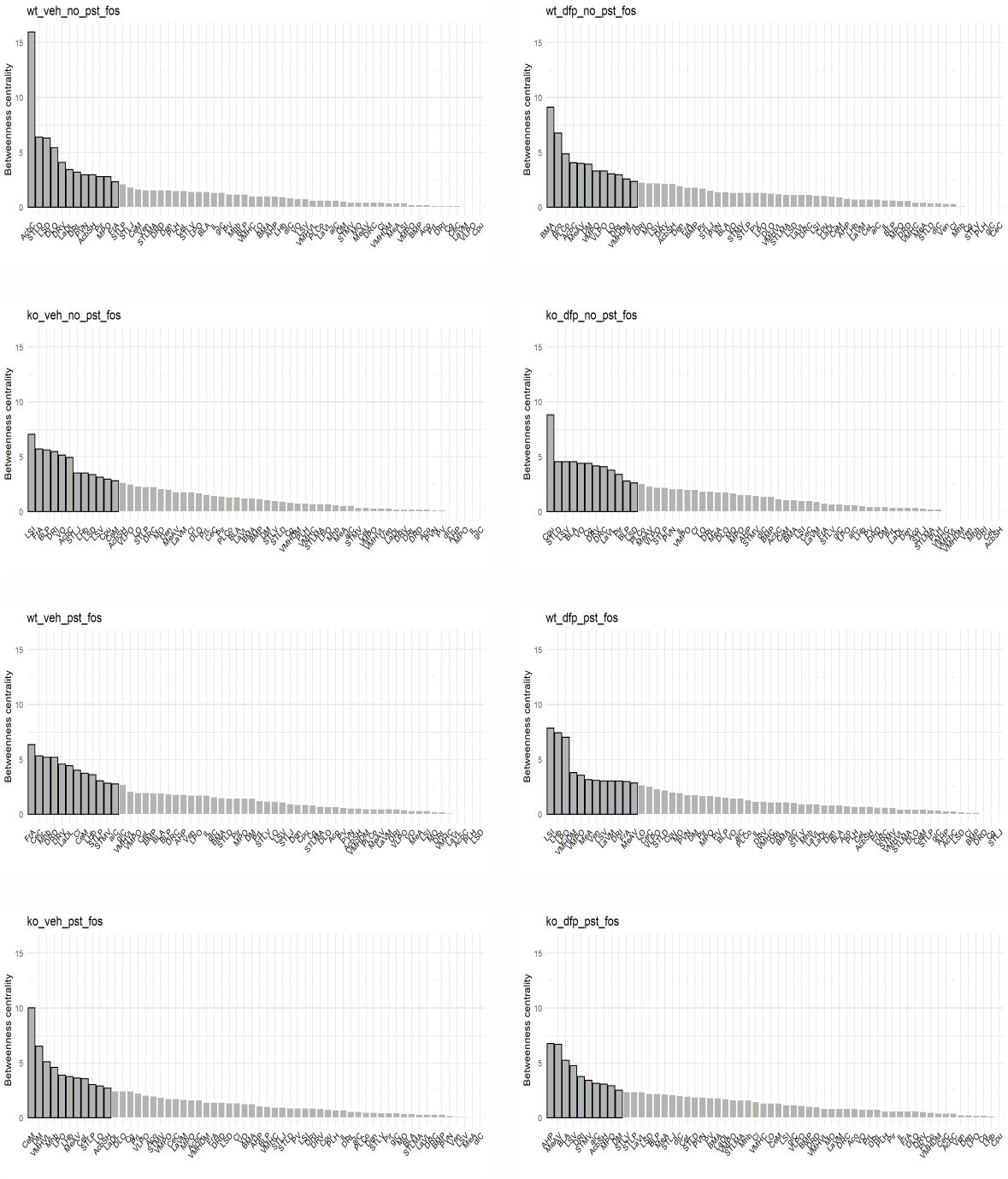

Supplement: Supplementary file 4 — Supplementary file4 (JPG 238 KB) [file 13311_2022_1257_MOESM4_ESM.jpg]
