# Supplementary material for: Novel Antidepressant-Like Properties of the Iron Chelator Deferiprone in a Mouse Model of Depression
Source: Neurotherapeutics. 2022 Jul 21;19(5):1662–85. doi: 10.1007/s13311-022-01257-0 (PMC9606181; doi:10.1007/s13311-022-01257-0)
Supplement: Supplementary file 5 — Supplementary file5 (DOCX 818 KB) [file 13311_2022_1257_MOESM5_ESM.docx]

**Novel antidepressant-like properties of the iron chelator deferiprone in a mouse model of depression**

**SUPPLEMENTARY MATERIALS**

***SUPPLEMENTARY METHODS***

**Light-dark box**

The light-dark box (LDB) is an apparatus which consists of a photo-beam activity chamber (26 x 26 x 38 cm) (Coulburn Instruments, Pennsylvania, USA). The chamber is split into two compartments by a black Plexiglass insert. The black insert includes a small 4 x 4 cm opening which allows the mouse to explore between the dark and light compartments. The light compartment is lit brightly at 750 lux. Mice are placed in the dark compartment 1-hour after injection of vehicle or deferiprone facing away from the opening into the light compartment and allowed to freely explore for 10 minutes. The amount of time spent in each compartment and entrance to each compartment is automatically recorded by the infrared beams of the chamber. Less time spent in the light compartment is indicative of an anxiety-like phenotype.

***SUPPLEMENTARY DATA***

**
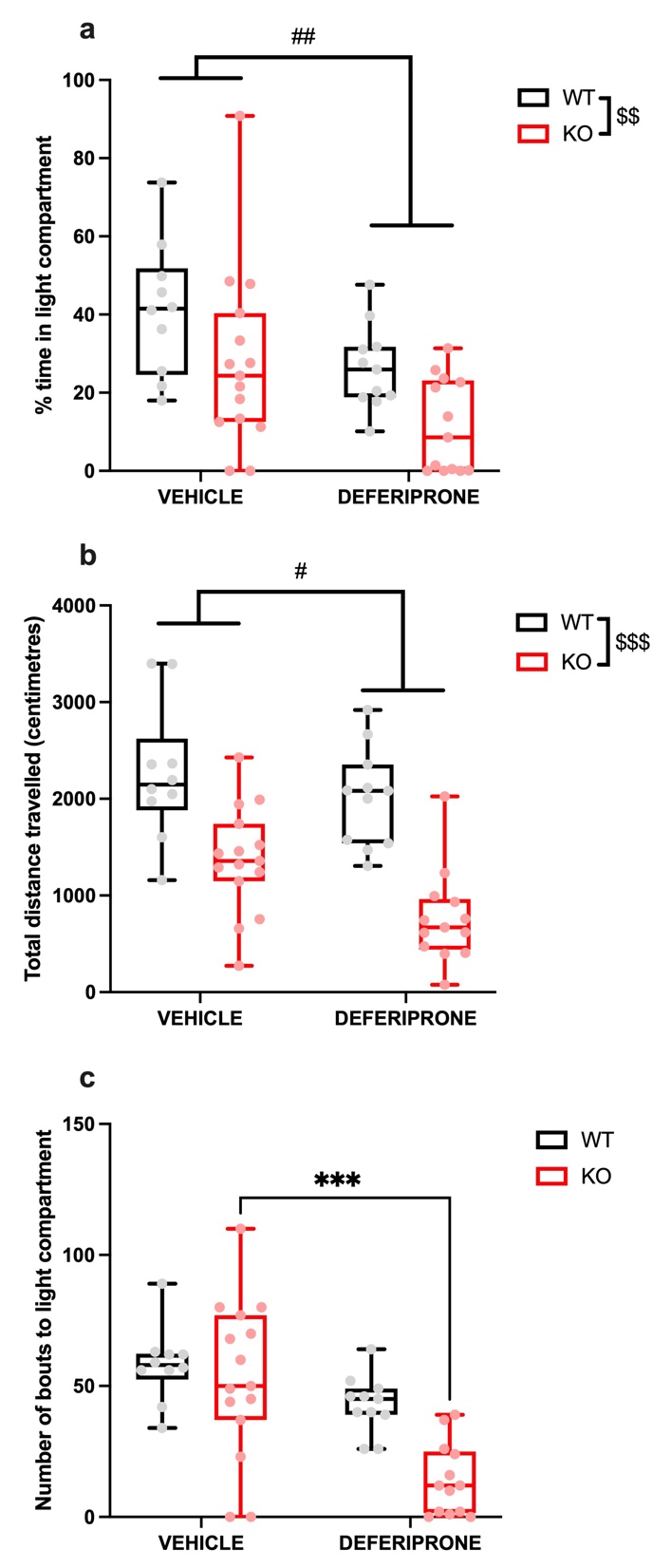
**

**Supplementary Fig. 1** Acute deferiprone treatment had an anxiogenic-like effect in the light-dark box test irrespective of genotype. Deferiprone reduced time spent in the light compartment (treatment - F_(1,45)_ = 10.10, p<0.01) **(a)** and total distance travelled (treatment - F_(1,45)_ = 7.07, p<0.05) **(b)** during the test irrespective of genotype. Deferiprone selectively reduced the number of bouts to the light compartment in 5-HTT KO mice (genotype x treatment interaction – F_(1,45)_ = 4.26, p<0.05; post-hoc – p<0.001) **(c)**. 2-way ANOVA; Bonferroni post-hoc. Data are expressed as median with interquartile range; whiskers represent min to max values. n=10-15. ***p<0.001 KO VEHICLE vs KO DEFERIPRONE, ##p<0.01;p<0.05 VEHICLE vs DEFERIPRONE, $$$p<0.001;$$p<0.01 WT vs KO. WT = wild-type; KO = knock-out.

**
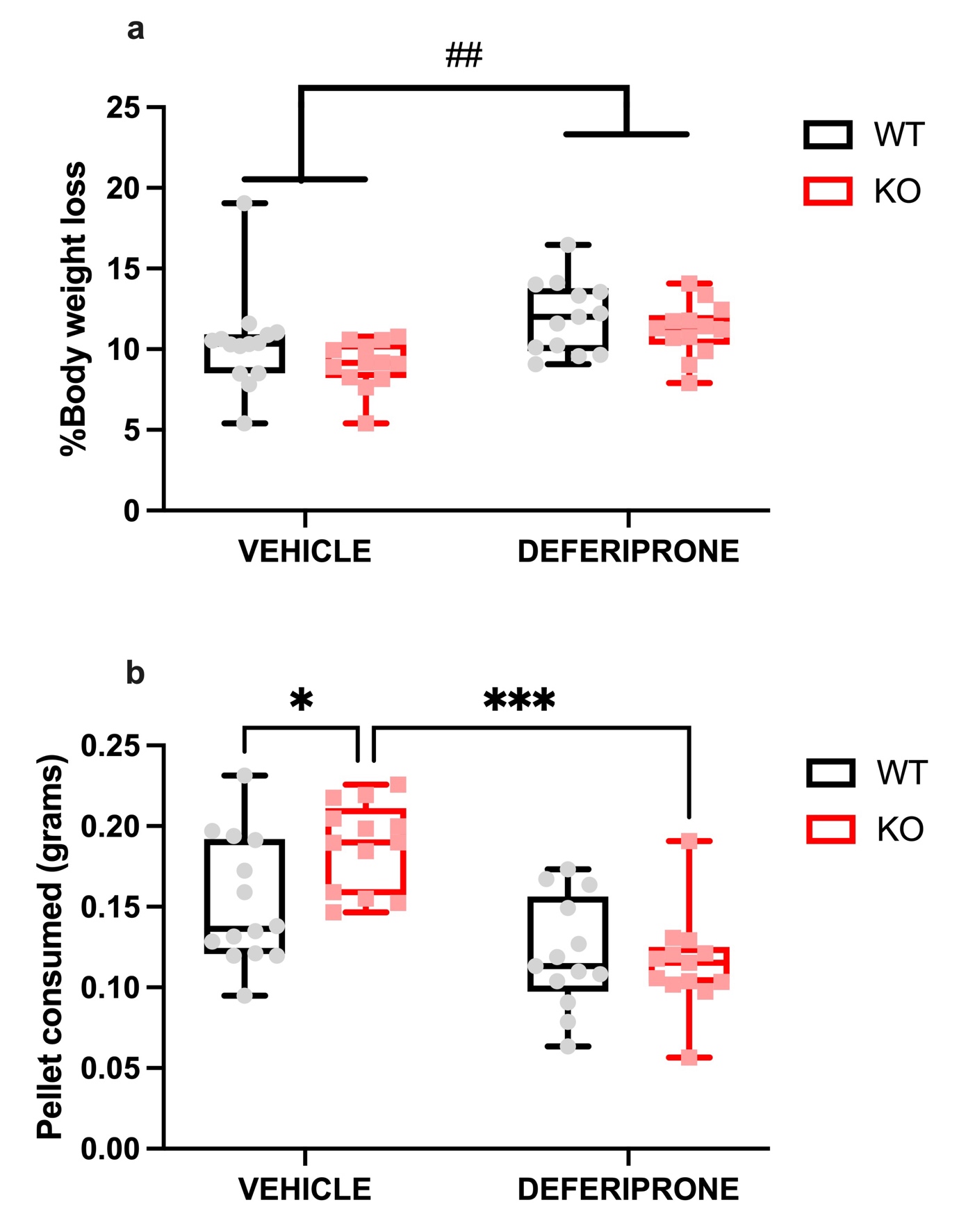
**

**Supplementary Fig. 2** Weight loss during the fasting period and pellet consumption following the novelty-suppressed feeding test. Mice which were to receive deferiprone had greater weight loss in the fasting period compared to vehicle controls (treatment – F_(1,49)_ = 9.77, p<0.05) **(a)**. 5-HTT KO mice which received deferiprone had reduced pellet consumption in the home cage following the novelty-suppressed feeding test (genotype x treatment interaction – F_(1,49)_ = 5.16, p<0.05; KO VEHICLE vs KO DEFERIPRONE post-hoc – p<0.001; WT VEHICLE vs KO VEHICLE post-hoc – p<0.05) **(b)**. 2-way ANOVA; Bonferroni post-hoc. Data are expressed as median with interquartile range; whiskers represent min to max values. n=13-14. *p<0.05 WT VEHICLE vs KO VEHICLE;***p<0.001 KO VEHICLE vs KO DEFERIPRONE, ##p<0.01VEHICLE vs DEFERIPRONE. WT = wild-type; KO = knock-out.

**Supplementary Table 1** Quantity of key metals following acute deferiprone treatment in various brain regions and peripheral blood. Values as are expressed as μg/g and μmol/L for brain regions and blood levels, respectively. When running a 2-way ANOVA, we found a main effect of deferiprone on Sodium levels in the prefrontal cortex (F_(1,44)_ = 4.17, p<0.05) and a significant effect of genotype on Zinc levels in blood (F_(1,44)_ = 5.06, p<0.05). #p<0.05 VEHICLE vs DEFERIPRONE, $p<0.05 WT vs KO. Values are presented as the mean ± SEM, n = 9-14 per group. WT = wild-type; KO = 5-HTT knock-out.

|  | WT Vehicle | KO Vehicle | WT Deferiprone | KO Deferiprone |
| --- | --- | --- | --- | --- |
|  | **Prefrontal cortex** | | | |
| Sodium | 1014.61 ± 35.34 | 960.34 ± 23.00 | 1035.38 ± 35.62^#^ | 1073.97 ± 36.97^#^ |
| Magnesium | 142.23 ± 5.67 | 134.86 ± 3.45 | 140.87 ± 4.95 | 144.02 ± 2.72 |
| Aluminium | 0.32 ± 0.050 | 0.36 ± 0.11 | 0.31 ± 0.047 | 1.59 ± 1.15 |
| Manganese | 0.29 ± 0.012 | 0.28 ± 0.0072 | 0.29 ± 0.012 | 0.30 ± 0.0077 |
| Copper | 3.77 ± 0.19 | 3.53 ± 0.15 | 3.75 ± 0.21 | 3.78 ± 0.17 |
| Zinc | 13.19 ± 0.45 | 12.63 ± 0.33 | 13.01 ± 0.37 | 13.47 ± 0.27 |
| Selenium | 0.074 ± 0.012 | 0.075 ± 0.0081 | 0.085 ± 0.011 | 0.10 ± 0.0097 |
|  | **Striatum** | | | |
| Sodium | 1211.78 ± 132.32 | 1122.85 ± 76.85 | 1250.74 ± 154.11 | 1214.17 ± 101.20 |
| Magnesium | 125.60 ± 12.74 | 115.45 ± 8.44 | 121.011 ± 12.13 | 118.57 ± 9.83 |
| Aluminium | 0.78 ± 0.066 | 0.88 ± 0.14 | 1.41 ± 0.58 | 0.84 ± 0.13 |
| Manganese | 0.39 ± 0.035 | 0.36 ± 0.034 | 0.40 ± 0.059 | 0.35 ± 0.025 |
| Copper | 9.17 ± 1.17 | 9.89 ± 0.99 | 7.57 ± 0.78 | 10.17 ± 1.16 |
| Zinc | 11.42 ± 1.17 | 10.81 ± 0.62 | 11.58 ± 1.63 | 11.30 ± 0.95 |
| Selenium | 0.14 ± 0.020 | 0.10 ± 0.014 | 0.12 ± 0.019 | 0.13 ± 0.014 |
|  | **Brainstem** | | | |
| Sodium | 1209.94 ± 25.92 | 1211.75 ± 11.72 | 1243.94 ± 23.14 | 1261.22 ± 20.25 |
| Magnesium | 132.017 ± 3.45 | 131.00 ± 1.95 | 129.34 ± 2.63 | 130.26 ± 1.33 |
| Aluminium | 0.16 ± 0.022 | 0.36 ± 0.12 | 0.15 ± 0.016 | 0.18 ± 0.029 |
| Manganese | 0.44 ± 0.016 | 0.41 ± 0.0094 | 0.40 ± 0.019 | 0.41 ± 0.012 |
| Copper | 3.18 ± 0.12 | 2.93 ± 0.099 | 3.056 ± 0.17 | 3.052 ± 0.081 |
| Zinc | 7.83 ± 0.18 | 7.80 ± 0.15 | 7.65 ± 0.17 | 7.78 ± 0.088 |
| Selenium | 0.15 ± 0.0082 | 0.14 ± 0.0062 | 0.14 ± 0.0062 | 0.16 ± 0.0042 |
|  | **Blood** | | | |
| Sodium | 86209.16 ± 916.63 | 80136.48 ± 2934.30 | 83665.45 ± 1545.65 | 81308.97 ± 3040.14 |
| Magnesium | 1510.35 ± 38.28 | 1504.26 ± 36.91 | 1529.98 ± 31.65 | 1577.49 ± 46.85 |
| Aluminium | 3.17 ± 0.43 | 3.40 ± 0.65 | 4.80 ± 1.35 | 2.45 ± 0.27 |
| Manganese | 0.48 ± 0.027 | 0.47 ± 0.043 | 0.52 ± 0.044 | 0.53 ± 0.051 |
| Copper | 5.91 ± 0.13 | 6.15 ± 0.17 | 6.31 ± 0.29 | 6.46 ± 0.25 |
| Zinc | 43.83 ± 1.026 | 49.59 ± 2.77^$^ | 45.27 ± 1.23 | 49.72 ± 2.57^$^ |
| Selenium | 6.23 ± 0.27 | 6.48 ± 0.26 | 6.31 ± 0.22 | 6.95 ± 0.34 |


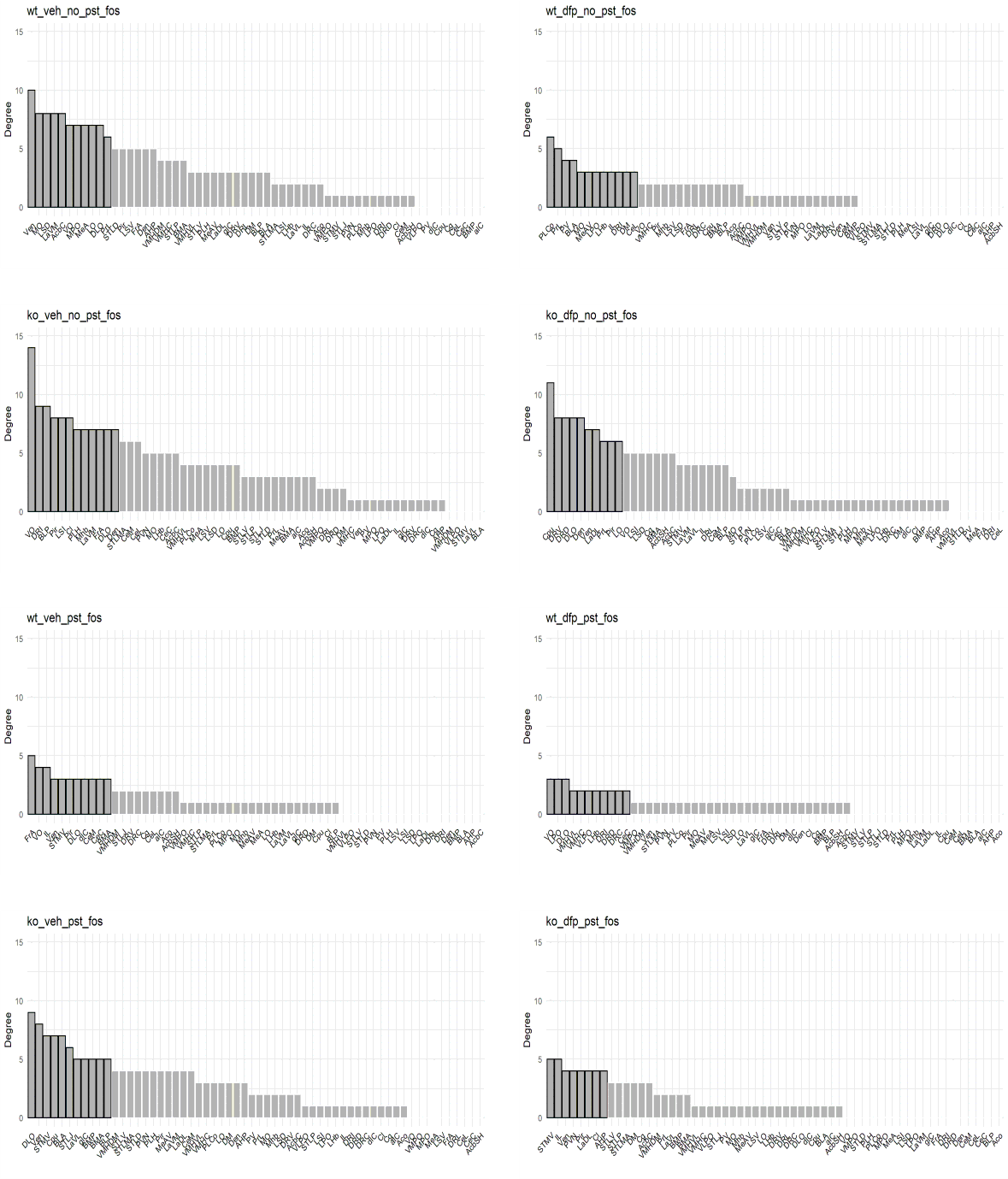


**Supplementary Fig. 3** Ranked number of significant degrees following deferiprone and following swim stress exposure in WT and 5-HTT KO mice

The regions are ranked in descending order with the 80^th^ percentile regions in bold. WT = wild-type; KO = 5-HTT KO; DFP = deferiprone.


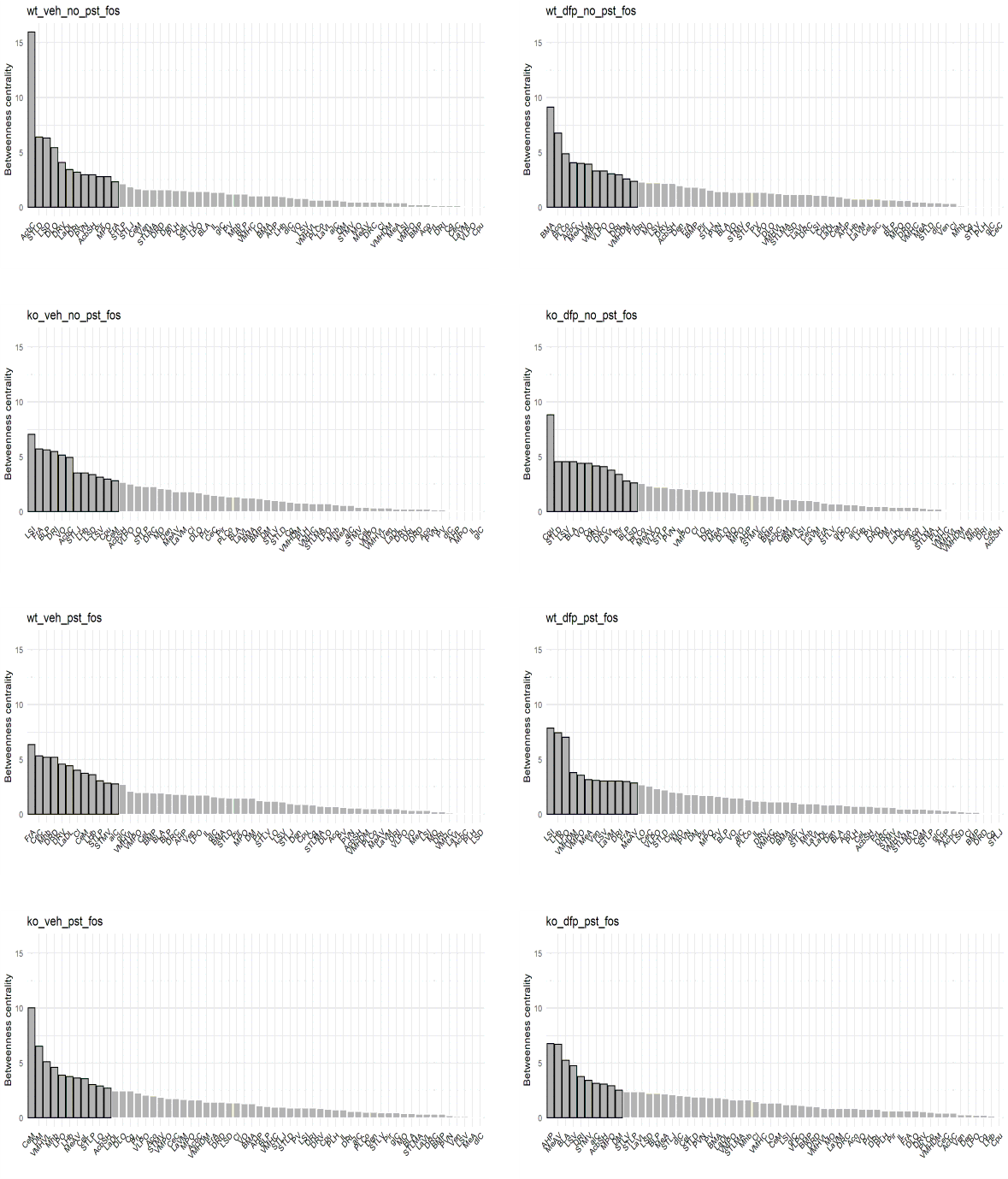


**Supplementary Fig. 4** Ranked ‘betweenness centrality’ following deferiprone and swim stress (PST) exposure in WT and 5-HTT KO mice

The regions are ranked in descending order with the 80^th^ percentile regions in bold. WT = wild-type; KO = 5-HTT KO; DFP = deferiprone.

**Supplementary Table 2** List of brain regions in which c-Fos expression was quantified and the corresponding abbreviation and number on correlation heatmap

| Region | Abbreviation | Number |
| --- | --- | --- |
| Prefrontal cortex  Prelimbic cortex  Infralimbic cortex  Frontal association cortex  Medial orbital cortex  Ventral orbital cortex  Lateral orbital cortex  Dorsolateral orbital cortex | PrL  IL  FrA  MO VO  LO DLO | 1  2  3  4  5  6  7 |
| Cerebral cortex  Cingulate cortex  Claustrum  Agranular insular cortex  Dysgranular insular cortex  Granular insular cortex | Cg  Cl  aIC  dIC  gIC | 8  9  10  11  12 |
| Lateral septum  Lateral septum, dorsal  Lateral septum, ventral  Lateral septum, intermediate | LSD  LSV  LSI | 13  14  15 |
| Hypothalamus  Paraventricular hypothalamus  Anterior hypothalamic area  Peduncular part of lateral hypothalamus  Dorsomedial hypothalamus  Ventromedial hypothalamus, ventrolateral  Ventromedial hypothalamus, dorsomedial  Ventromedial hypothalamus, central  Ventrolateral preoptic hypothalamus  Ventromedial preoptic hypothalamus  Medial preoptic hypothalamus  Lateral preoptic hypothalamus | PVN  AHP  PLH  DM  VMHVL  VMHDM  VMHC  VLPO  VMPO  MPO  LPO | 16  17  18  19  20  21  22  23  24  25  26 |
| Striatum  Nucleus accumbens, core  Nucleus accumbens, shell  Caudate putamen | AcbC  AcbSH  Cpu | 27  28  29 |
| Amygdaloid regions  Medial amygdala, anteroventral  Medial amygdala, anterodorsal  Anterior cortical amygdala area  Posterolateral cortical amygdala area  Piriform cortex  Basomedial amygdala  Dorsal endopiriform claustrum  Ventral endopiriform claustrum  Central amygdala, capsular  Central amygdala, lateral  Central amygdala, medial  Lateral amygdala, dorsolateral  Lateral amygdala, ventrolateral  Lateral amygdala, ventromedial  Basolateral amygdala, posterior  Basolateral amygdala, anterior  Basomedial amygdala, posterior | MeAV  MeA  Aco  PLCO  Pir  BMA  Den  Ven  CeC  CeL  CeM  LaDL  LaVL  LaVM  BLP  BLA  BMP | 30  31  32  33  34  35  36  37  38  39  40  41  42  43  44  45  46 |
| Bed nucleus of stria terminalis  BNST, medial, ventral  BNST, lateral, ventral  BNST, lateral, posterior  BNST, lateral, juxtacapsular  BNST, lateral, dorsal  BNST, medial, anteromedial | STMV  STLV  STLP  STLJ  STLD  STLMA | 47  48  49  50  51  52 |
| Thalamus  Medial habenula  Lateral habenula  Paraventricular thalamus | Mhb  Lhb  PV | 53  54  55 |
| Dorsal raphe  Dorsal raphe, dorsal  Dorsal raphe, ventral  Dorsal raphe, intrafascular  Dorsal raphe, lateral  Dorsal raphe, caudal | DRD  DRV  DRI  DRL  DRC | 56  57  58  59  60 |
